# Common Functional Correlates of Head-Strike Behavior in the Pachycephalosaur *Stegoceras validum* (Ornithischia, Dinosauria) and Combative Artiodactyls

**DOI:** 10.1371/journal.pone.0021422

**Published:** 2011-06-28

**Authors:** Eric Snively, Jessica M. Theodor

**Affiliations:** 1 Department of Mechanical Engineering, Ohio University, Athens, Ohio, United States of America; 2 Department of Biological Sciences, University of Calgary, Calgary, Alberta, Canada; Utah State University-College of Eastern Utah, United States of America

## Abstract

**Background:**

Pachycephalosaurs were bipedal herbivorous dinosaurs with bony domes on their heads, suggestive of head-butting as seen in bighorn sheep and musk oxen. Previous biomechanical studies indicate potential for pachycephalosaur head-butting, but bone histology appears to contradict the behavior in young and old individuals. Comparing pachycephalosaurs with fighting artiodactyls tests for common correlates of head-butting in their cranial structure and mechanics.

**Methods/Principal Findings:**

Computed tomographic (CT) scans and physical sectioning revealed internal cranial structure of ten artiodactyls and pachycephalosaurs *Stegoceras validum* and *Prenocephale prenes*. Finite element analyses (FEA), incorporating bone and keratin tissue types, determined cranial stress and strain from simulated head impacts. Recursive partition analysis quantified strengths of correlation between functional morphology and actual or hypothesized behavior. Strong head-strike correlates include a dome-like cephalic morphology, neurovascular canals exiting onto the cranium surface, large neck muscle attachments, and dense cortical bone above a sparse cancellous layer in line with the force of impact. The head-butting duiker *Cephalophus leucogaster* is the closest morphological analog to *Stegoceras*, with a smaller yet similarly rounded dome. Crania of the duiker, pachycephalosaurs, and bighorn sheep *Ovis canadensis* share stratification of thick cortical and cancellous layers. *Stegoceras*, *Cephalophus*, and musk ox crania experience lower stress and higher safety factors for a given impact force than giraffe, pronghorn, or the non-combative llama.

**Conclusions/Significance:**

Anatomy, biomechanics, and statistical correlation suggest that some pachycephalosaurs were as competent at head-to-head impacts as extant analogs displaying such combat. Large-scale comparisons and recursive partitioning can greatly refine inference of behavioral capability for fossil animals.

## Introduction

Many animals strike with their heads at conspecifics, in ritualized flank-butting, head-to-head shoving matches and head-butting combat. Correlates cited for head-butting in modern ungulates include cranial sinuses [1] that form strut-perfused osseous domes above the brain, and secondary correlates include neurovascular canals supplying a protective keratin covering on the skull surface [2]. In addition to colliding with their horns (which spreads the impact in dual horn-horn contacts [3]), bighorn sheep (*Ovis canadensis*) vigorously impact each other on the apices of their heads between the horn cores [4]. Duikers (Cephalophinae) are small bovids with thick, rounded frontals, which they use in intraspecific head-to-head impacts [5], [6]. Similarly dome-shaped crania of pachycephalosaurian dinosaurs have been hypothesized as appropriate for head- or flank-butting, but internal histology appears to contradict such capability in young and old individuals [7] unless a thick keratinous covering protected the osseous dome [8].

Dome function in pachycephalosaurs has been controversial, with trabeculae within the dome interpreted as developmental traces inconsistent with head-butting [7], or as structures that would halt or absorb strain during collisions [9]. A highly vascular cancellous zone [7] undoubtedly sped the development and growth of pachycephalosaur domes. However, quantitative tests have supported a complementary energy-absorbing role for trabeculae. Farke [1] determined that trabeculae within cranial sinuses of goats would better dissipate strain than sinuses alone, similar to what Snively and Cox [8] found for cancellous regions of some pachycephalosaur domes. Maity and Tekalur [10] corroborated this phenomenon in bighorn sheep, despite a different loading pattern.

We use CT (computed tomographic) scanning and finite element analysis (FEA) to compare structural capabilities of crania in head-striking artiodactyls (the duiker *Cephalophus leucogaster*, musk ox *Ovibos moschatus* and giraffe *Giraffa camelopardalis* [6], [10], [11], [12], [13]) and a possibly analogous combatant, the pachycephalosaur *Stegoceras validum*. As controls we examine specimens of other artiodactyls that engage in a spectrum of combative behaviors. Bighorn sheep [*Ovis canadensis*) butt heads, and regress in horn-horn contact as these ornaments take a greater display function in older rams [4]. Male pronghorn *Antilocapra americana* collide with cranial ornaments but do not butt heads [14], whereas neither behavior occurs in female elk (wapiti) *Cervus canadensis*, peccary *Tayassu tajacu* and llama (*Lama glama*). Expanding on previous studies of pachycephalosaur crania, we perform 3D finite element analysis (FEA) of simulated head impacts with models based on CT scans.

These methods test three main hypotheses and predictions. First, we test the prediction that like some pachycephalosaurs [8], head-butting artiodactyls will have a deep layer of cancellous bone beneath dense compact bone. Second, we extend Farke's hypothesis for goats ([1], [5], and references therein), that frontal sinuses with trabeculae would dissipate strain, to other head-butting bovids. The morphology of soft tissue covering pachycephalosaur domes is unknown, and we test the effects of different-shaped keratin pads by applying concentrated and broad forces to the dome. Finally, we examine whether *Stegoceras*, musk ox and duiker cephalic structures would experience similar stress levels under similar impact loads.

### Structural terminology and interpretation of finite element results

In the following discussions, ultimate stress refers to the material's strength (breaking point), and yield stress is that of permanent deformation (beginning the inelastic portion of the stress-strain curve). Ultimate stress is usually higher than yield stress, and results in full breakage or crushing of a structure or its constituents. A brittle material, with little porosity to dissipate energy of cracking, often has similar strength and yield stresses. Bone has both a brittle mineralized component and ductile, flexible collagen, whereas keratin is highly ductile. Safety factor refers to ultimate or yield stress (or strain) divided by the experienced value.

When FEA reveals safety factors under given loads, we can predict and compare forces necessary to break the structures. A cranial element with a higher safety factor can experience higher magnitudes of impact force before it breaks, which would potentially damage critical soft tissues. (A classic example is a blow to the squamous portion of the temporal in humans, rupturing the middle meningeal artery and causing epidural hematoma.) The strain threshold of fatal soft tissue injury dictates the maximum force of a strike, which can be higher if extensive stiff and compliant hard tissues diminish impact stress and absorb strain energy. The current comparison quantifies relative capability for head-strikes of hard tissues of the head, a major step for circumscribing behavioral hypotheses. In the absence of trace evidence, inference of actual behavior of fossil animals requires novel phylogenetic and statistical methods.

### Correlations of functional morphology and behavior

A potentially useful method for inferring behavior is recursive partition analysis (RPA), an algorithm used to guide diagnoses of illness based on correlations between an affliction and its symptoms. Analogously to a patient with an unknown illness and presented symptoms, an extinct animal can be diagnosed for an unknown behavior based on morphological traits. Correlation strengths for animals with known behavior can test for likelihood of the behavior in an extinct taxon. Hieronymous and colleagues [2] applied RPA to examine strength of correlation between types of soft tissue and their osteological correlates. We use RPA to examine how well osteology, head shape, and FEA results correlate with behaviors in our examined taxa and several others (Table 2).

We also introduce an extension of RPA called correlate disruption, which examines how strongly adult *Stegoceras* morphology would suit it for combat relative to extant head-butting taxa. For an extant animal with strong linkages between structure and behavior, the strengths of these correlations will decrease when an incorrect behavior is assigned in RPA. We can test for a hypothetical behavior in an extinct animal by varying its behavioral assignment. If the alternate hypothesis changes correlation strength by the same amount as for the mis-assigned extant animal, the extinct animal can be inferred as an equivalently probable candidate for the behavior.

## Results

### Bone densities of Stegoceras and artiodactyl crania

**Figure 1:**
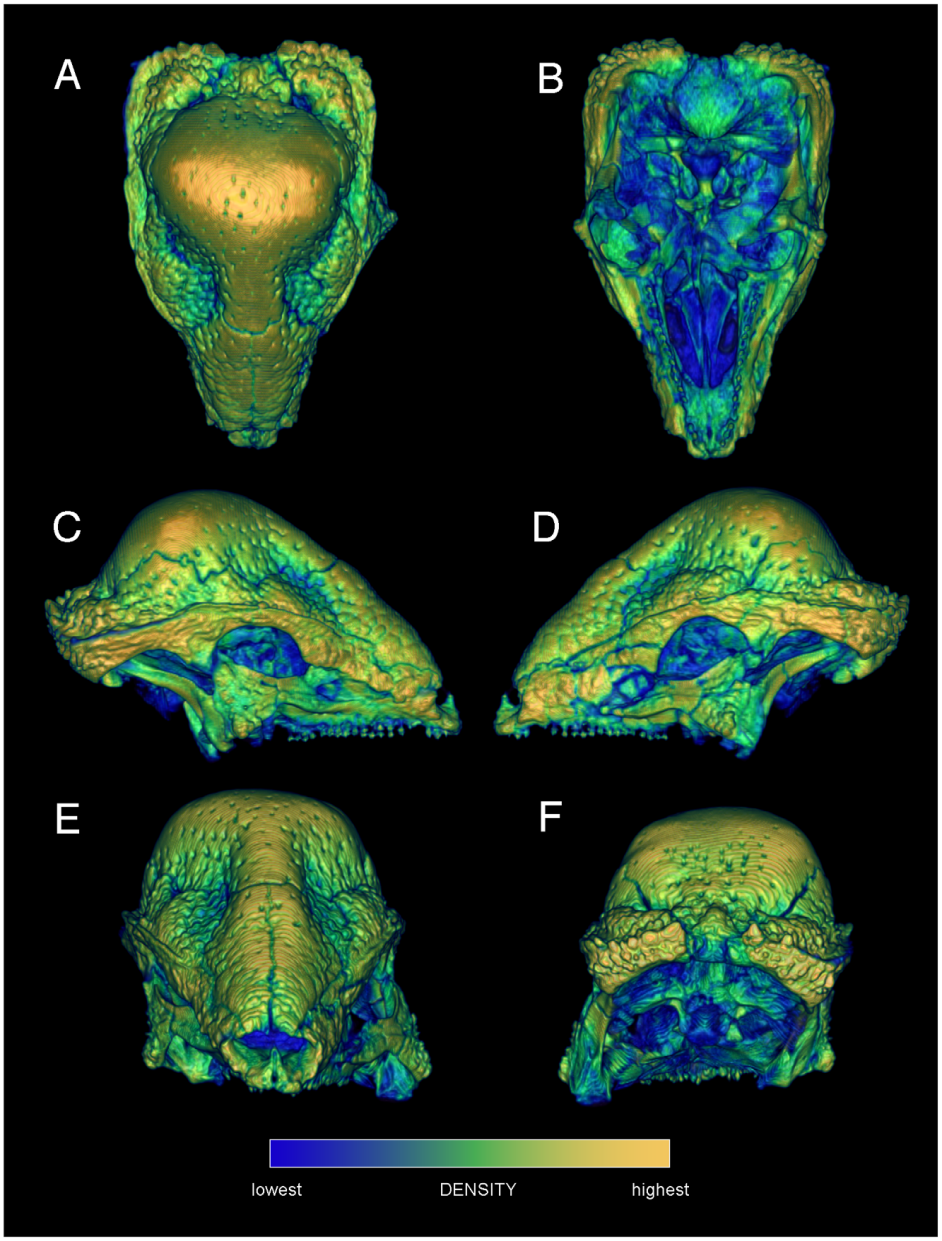
Relative surface densities of cranial bone in *Stegoceras validum* (UA 2). External densities of the cranium of *Stegoceras validum*, in dorsal (A), ventral (B), lateral (C, D), anterior (E) and posterior (F) views. Note high densities of cranial ornamentation, and numerous neurovascular canals (correlates of a keratinous pad) exiting onto the cranial roof.

**Figure 2:**
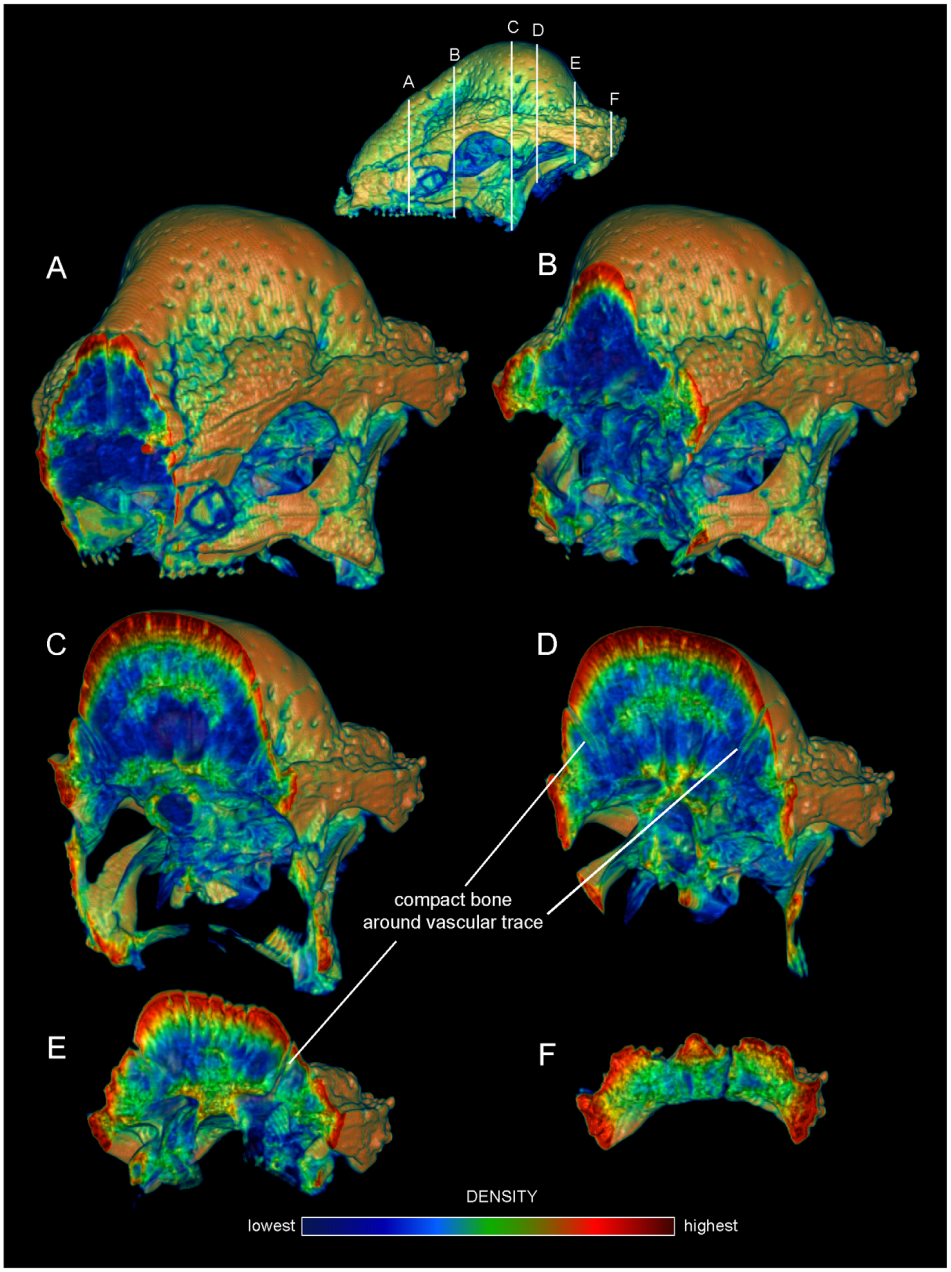
Internal densities of bone in *Stegoceras validum* (UA 2). Transverse CT sections from anterior (A) to posterior (F) through the cranium of *Stegoceras validum* (UA 2) seen in anterior oblique view. Inset CT reconstruction in lateral view (top) depicts section positions. Density and thickness of cortical bone increase towards the apex of the dome from the periphery, anteroposteriorly (B–D) and medially (C, D). Trabeculae radiate roughly perpencidular to the dome's outer surface, evident in the low-density (blue) region posterior to the orbit (B–E). Note that density of superficial bone may be inflated by beam hardening, but a dense, deep compact layer is definitively present. Dense compact bone (Hounsfield values of approximately 2000) surrounds presumed vascular traces, forming tubes that empty onto the dome surface; three of these are visible in D and E. These tubular structures recall struts within artiodactyl cranial sinuses.

**Figure 3:**
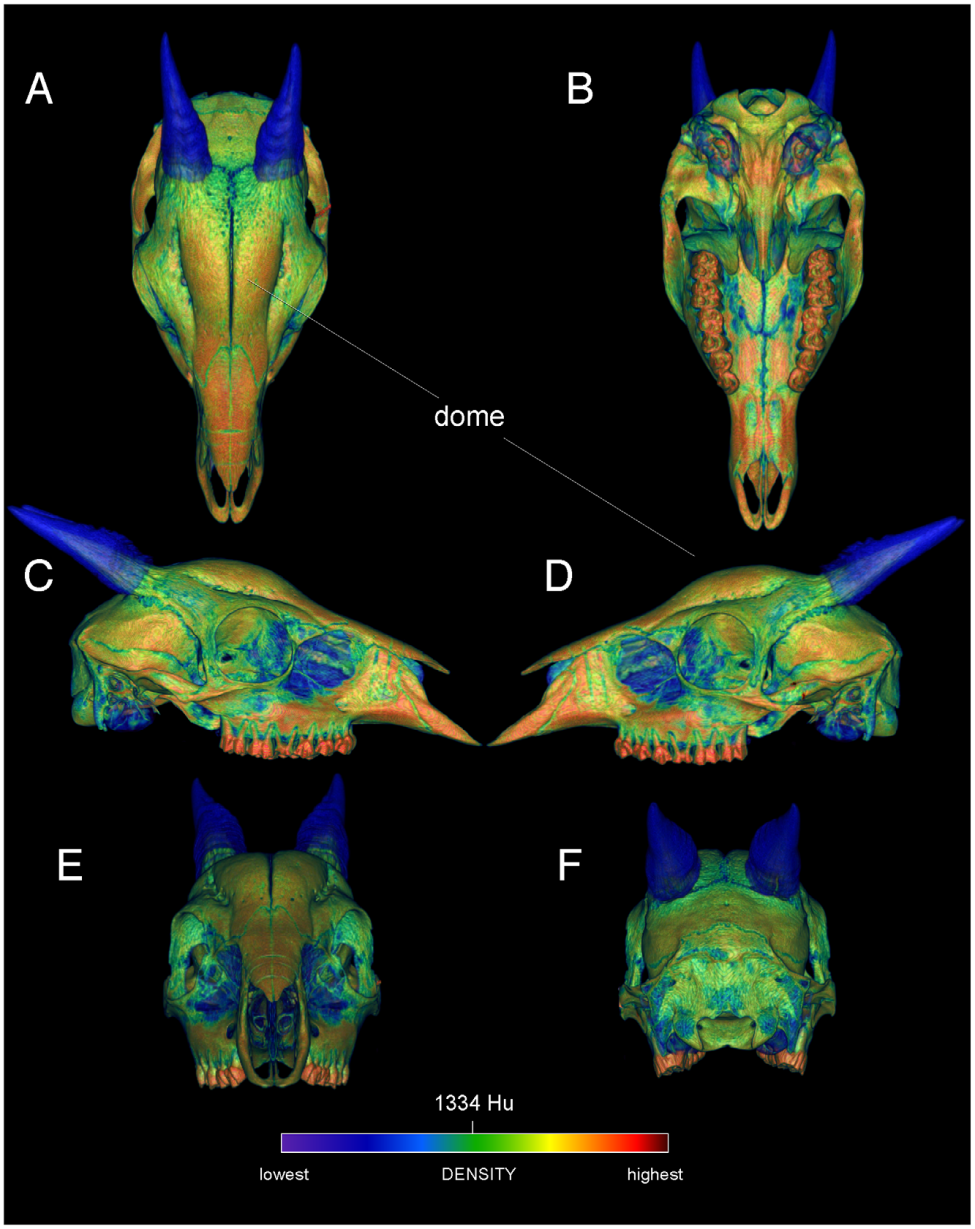
Surface densities of cranial bone in the duiker *Cephalophus leucogaster* (AMNH 52802). External cranial densities of the white-bellied duiker, in dorsal, ventral (A, B), right and left lateral (C, D), and anterior and posterior (E, F) views. Duikers collide with a rounded dome formed by thick frontals (the frontals are not fused, as in *Stegoceras*). The color scale is in Hounsfield units, centered at 1334 (water = 0). The horn sheaths are rendered as slightly transparent, to emphasize high densities of the horn cores; compare with the musk oxen (Figures 4, 5, 6).

**Figure 4:**
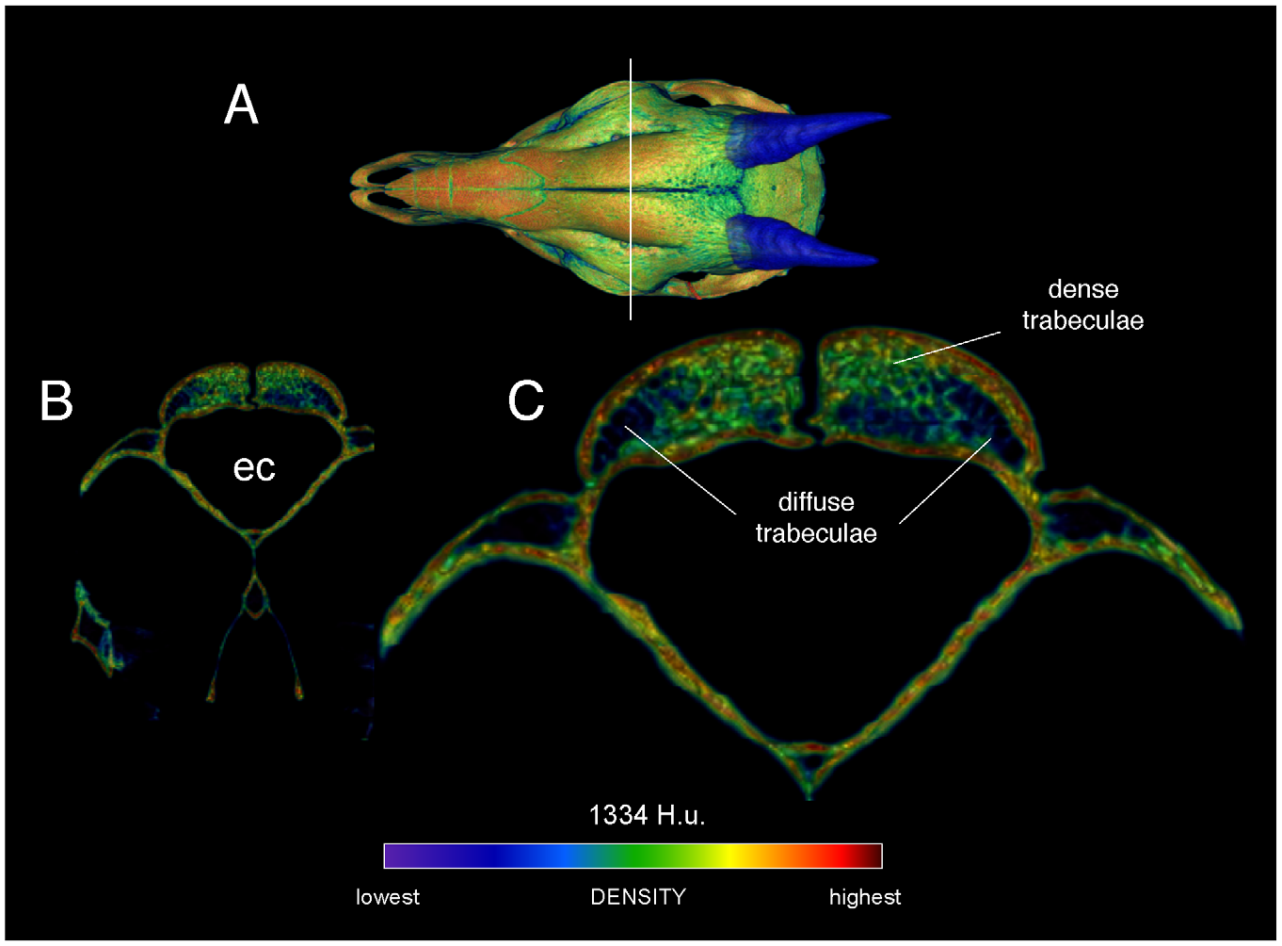
Densities of the frontal dome of *Cephalophus leucogaster* (AMNH 52802) in transverse section. A section through the posterior portion of the orbit and anterior region of the endocranial cavity (A, B) shows dense and diffuse trabecular bone (C) between bands of compact bone. Regions of compact bone are thinner than in *Stegoceras*, and larger trabeculae appear more robust (compare with Figure 2). The density color scale is the same as in Figure 3.

**Figure 5:**
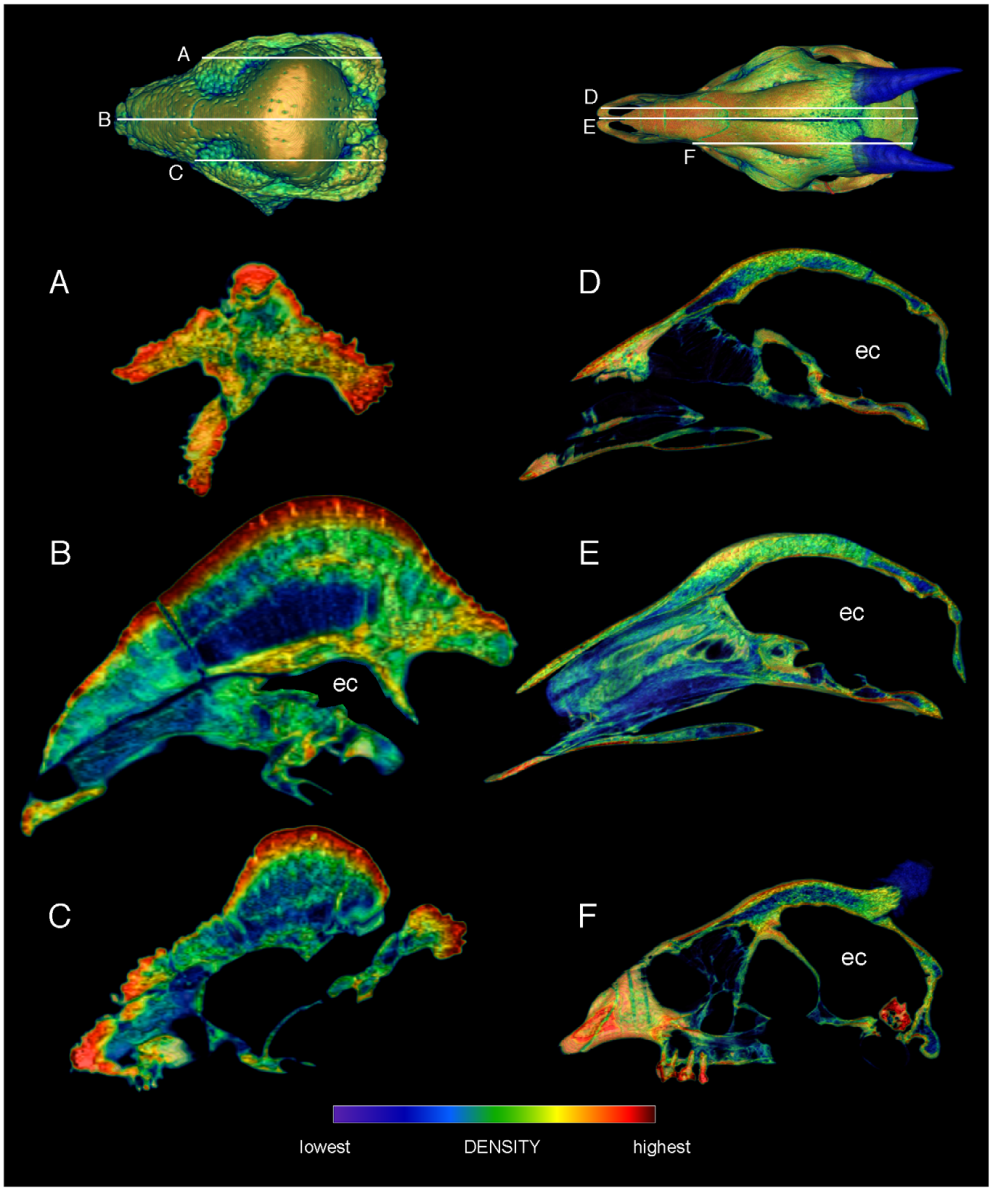
Comparison of sagittal-section densities in crania of *Stegoceras validum* (UA 2) and *Cephalophus leucogaster* (AMNH 52802). A, B, C. Sagittal sections through the cranium of *Stegoceras validum*, at positions shown in the dorsal view (top). D, E, F. Sections through the cranium of *Cephalophus leucogaster*, at positions shown in the dorsal inset (top). Note similar stratification of compact and cancellous layers in F, through the middle of the duiker's lobate dome, and B, through the center of the pachycephalosaur's dome. ec = endocranial cavity.

**Figure 6:**
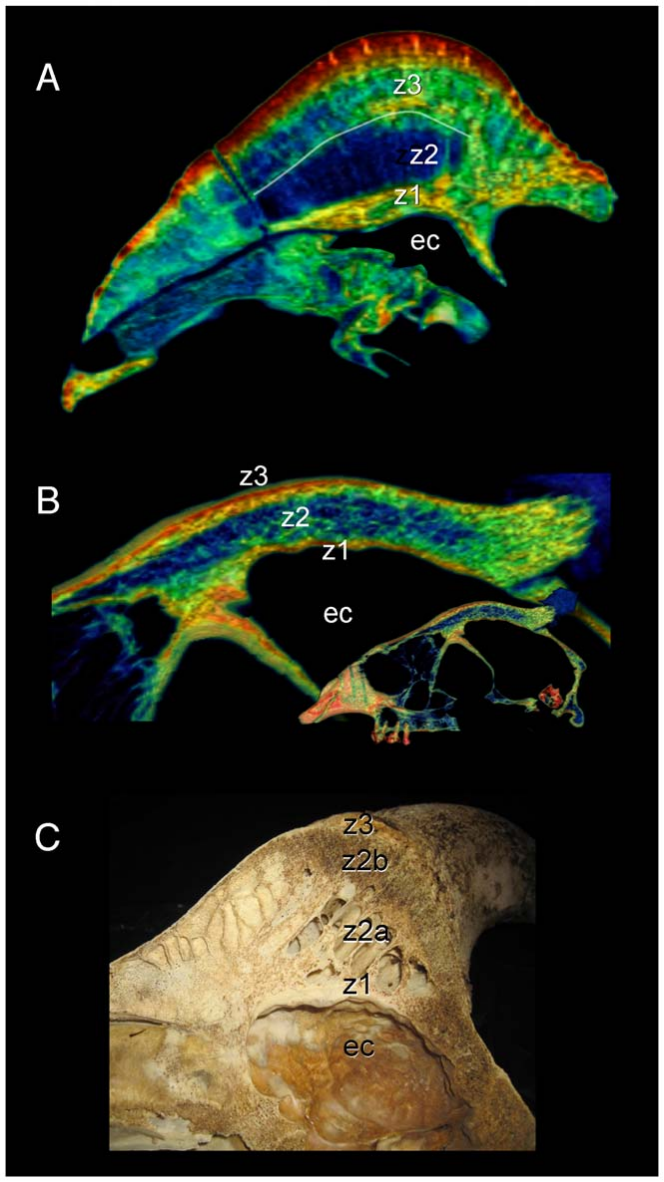
Comparison of dome structure in *Stegoceras* (UA 2), the duiker *Cephalophus* (AMNH 52802), and bighorn sheep *Ovis canadensis* (UCMZ). Midsagittal sections through the crania of *Stegoceras validum*, duiker *Cephalophus leucogaster*, and a bighorn sheep *Ovis canadensis* reveal similar dome structure. A. In the *Stegoceras* specimen, compact bone (z1 and z3: zones 1 and 3: [7]) occurs deep and superficial to a cancellous region (z2: zone 2: [7]). Moderately dense compact bone shows as a green band at the base of zone 3 (white line); note cancellous bone (blue) above the line in the anterior portion of this zone. B. *Cephalophus*. C. Similar stratification is evident in the sectioned *Ovis* cranium, with nearly identical zones of cancellous and compact bone broken by a ventral sinus.

**Figure 7:**
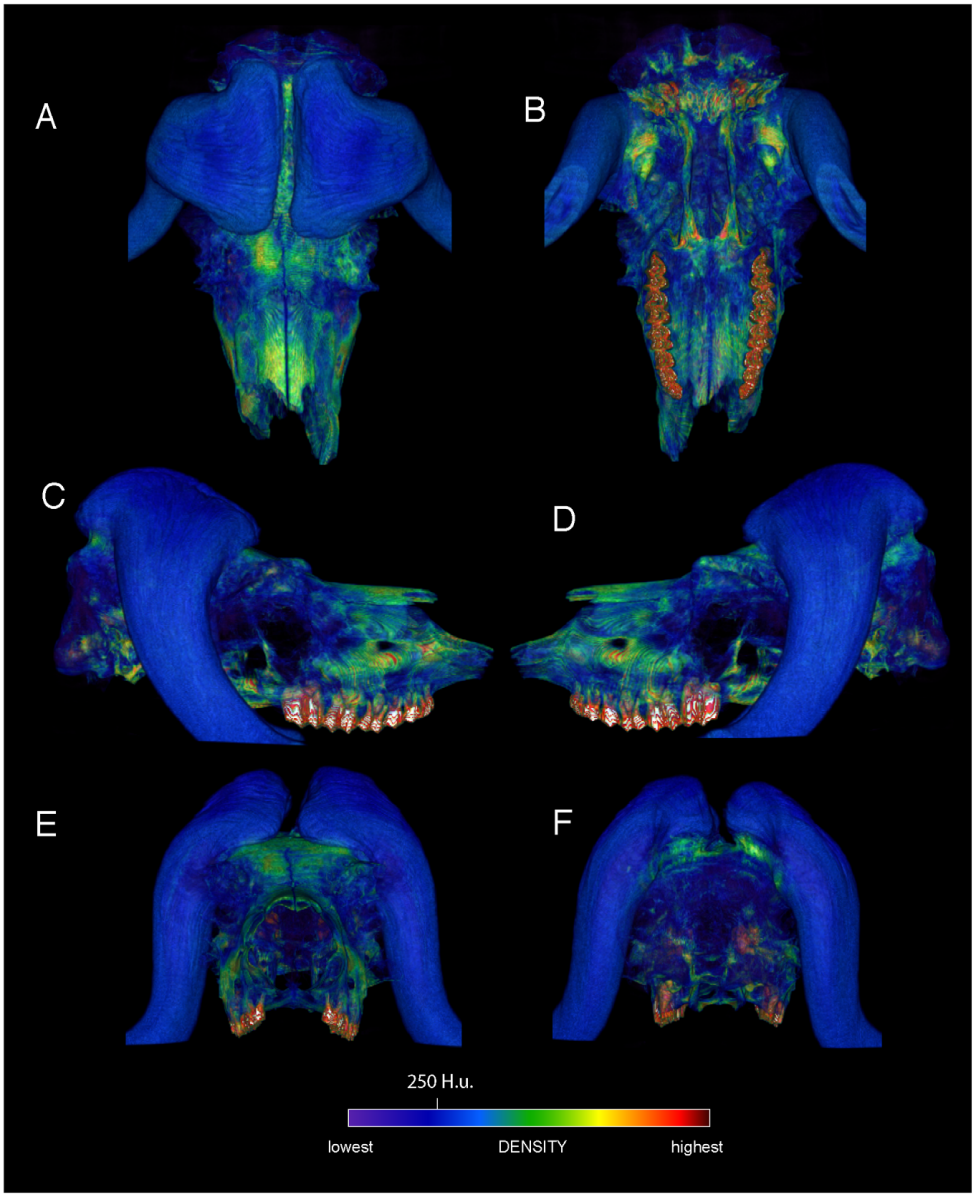
External cranial bone and horn densities in the adult musk ox (*Ovibos moschatus*: UCMZ M 1978.1.92). Densities of the cranium and horn sheaths in adult *Ovibos moschatus*, in dorsal (A), ventral (B), lateral (C, D), anterior (E) and posterior (F) views. Note bone higher bone densities than in the juvenile specimen (Figure 5), and the expanded keratin pads over the parietals.

**Figure 8:**
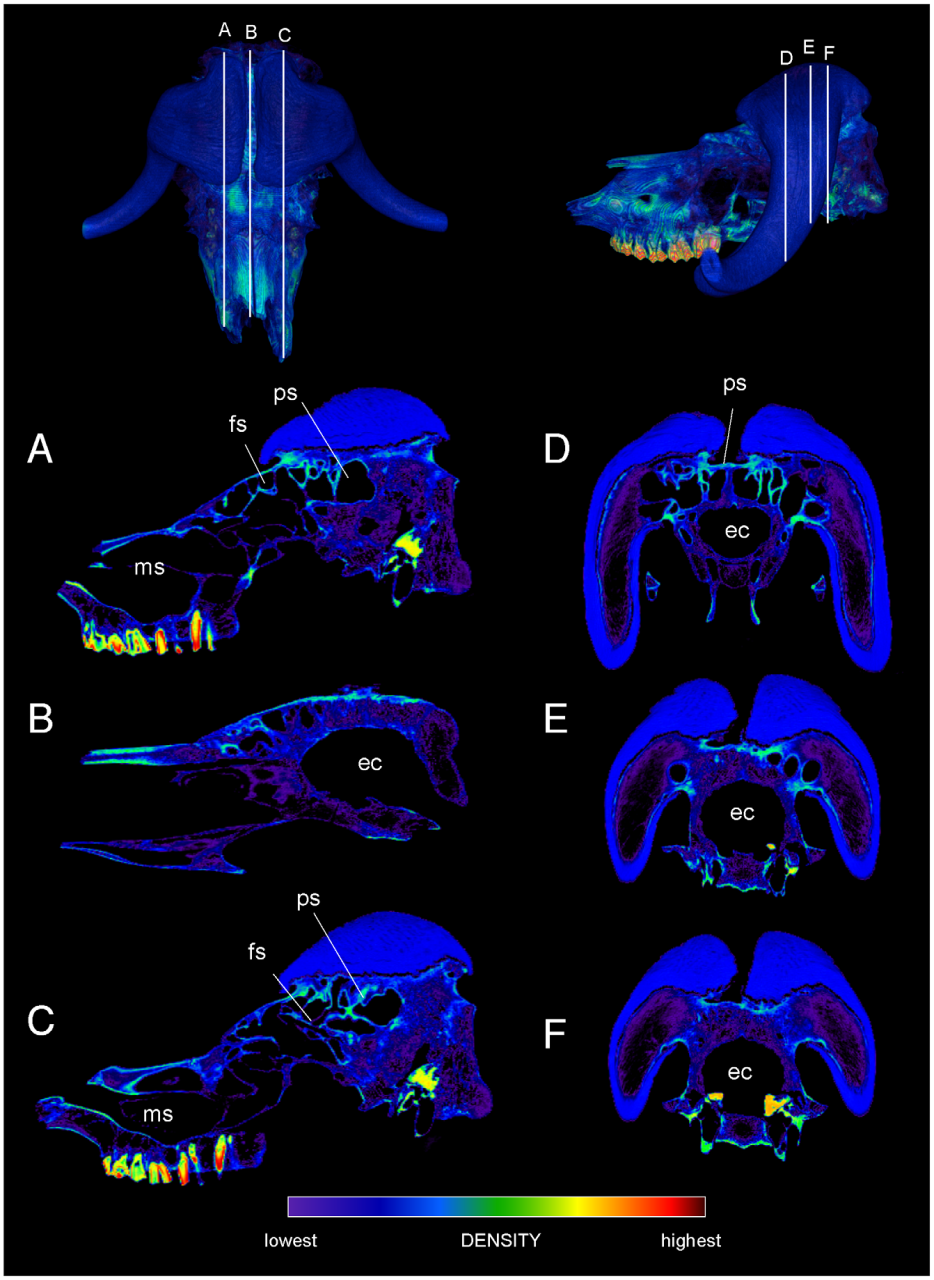
Internal densities of horn and bone in adult musk ox (*Ovibos moschatus* UCMZ M 1978.1.92). CT sections through the cranium of juvenile *Ovibos moschatus*. Dorsal and lateral CT renders show section position. A.–C. Right, mid-, and left sagittal sections show thick regions of trabeculae above the endocranial cavity, with a superficial layer of dense cortical bone over the apex of the brain (B). The most extensive cancellous bone occurs beneath the apeces of the horn sheaths, in line with the occipital condyles. A network of struts connects frontal and maxillary sinus regions (A and C). D.–F. Transverse sections from anterior to posterior show decreasing instances of struts and increasing cancellae as the horn sheaths become taller, and dense bone of the skull roof beneath the sheaths. Note the extent of trabecular bone between the sheaths and occipital condyles, in line with forces of head-butting impacts. Abbreviations: ec = endocranial cavity, fs = frontal sinus, ms = maxillary sinus, ps = parietal sinus.

**Figure 9:**
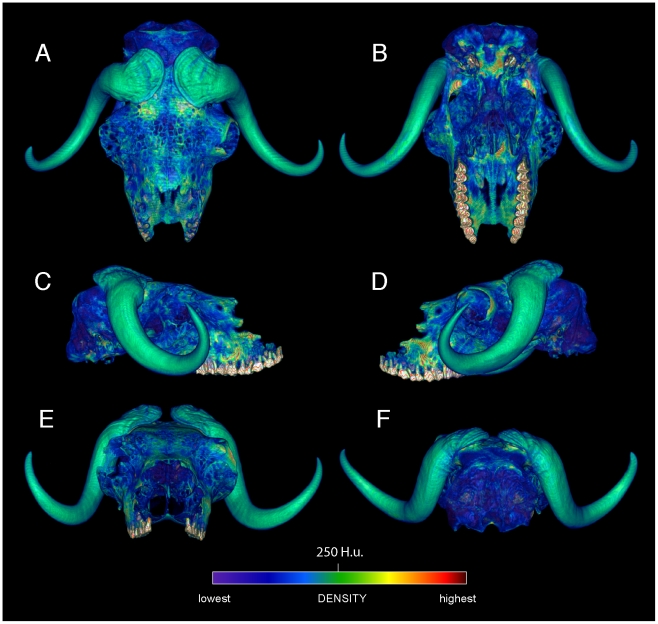
External densities of horn and bone in juvenile musk ox (UCMZ 1979.60). Densities of the cranium and horn sheaths in juvenile *Ovibos moschatus*, in dorsal (A), ventral (B), lateral (C, D), anterior (E) and posterior (F) views. Note higher densities anterior to the bases of the horns, and higher density of horn keratin than much of the cranial bone. The horns' keratin has yet to develop into a large pad above the parietals. Enamel densities are high and clipped out using this color scale.

Specimen numbers are listed in Table 1. Figures 1, 2, 3, 4, 5, 6, 7, 8, 9 depict external anatomy and densities from CT scans of *Stegoceras*, *Cephalophus*, and juvenile and adult *Ovibos*. Superficially the dome of *Stegoceras* is highly and uniformly dense (Figures 1), although CT beam hardening appears to increase Hounsfield values above those of the original bone (Figure 2). Densities of bone of the palate and occiput are substantially lower than those of the bone in the dome, supraorbital bones, and posterior ornaments (Figure 1 B and F). The external surface of the *Cepahlophus* dome (Figure 3) is denser than the horn-bearing portion of the frontals and the dorsal surface of the parietals, but not notably denser than the nasals or lateral portion of the parietals. The proximal horn cores of *Cephalophus* are much denser than their keratin sheaths. In contrast, horn sheaths in *Ovibos* are dense compared with cranial cancellous bone (Figures 7, 8, 9). The keratin sheaths of the adult *Ovibos* are denser and larger than in the juvenile (Figures 7 and 9), and bone densities overall are greater. The median visualized density for the juvenile *Ovibos* had to be lowered to easily depict keratin versus bone; hence the tooth enamel density is clipped and the teeth appear white (Figure 9).

**Table 1:** Taxa and specimens examined, and forces applied for FEA.

	Specimen number	Skull length (m)	Force for FEA (N)
*Antilocapra americana* (pronghorn)	UCMZ M 1989.61	0.27	3672
*Cephalophus leucogaster* (duiker)	AMNH 52802	0.17	1360
*Cervus canadensis* (elk/wapiti)	UCMZ M 1986.54	0.42	n/a
*Giraffa camelopardalis* (giraffe)	UCMZ 1976.33	0.65	51233
*Lama glama* (llama)	UCMZ M 1987.5	0.31	5558
*Ovibos moschatus* (musk ox)	UCMZ 1979.60	0.34	n/a
*Ovibos moschatus* (musk ox)	UCMZ M 1978.1.92	0.41	12858
*Prenocephale prenes* (pachycephalosaur)	GI SPS, field number PJC2004.8)	incomplete	n/a
*Stegoceras validum* (pachycephalosaur)	UALVP 2	0.18	1360
*Tayassu tajacu* (peccary)	UCMZ 1975.279	0.25	n/a

Abbreviations: UCMZ: University of Calgary Museum of Zoology. AMNH: American Museum of Natural History. GI SPS: Geological Institute Section of Paleontology and Stratigraphy, People's Republic of Mongolia. PJC: Philip J. Currie field number. UALVP: University of Alberta Laboratory for Vertebrate Paleontology.

As evident in histological sections through mature pachycephalosaur domes [7], CT sections into the *Stegoceras* cranium reveal dense inner and outer compact bone and a less dense intermediate region (zones 1, 3 and 2, respectively, identified by Goodwin and Horner [7]). The outer zone of compacta becomes thicker towards the apex of the dome (Figure 2) from all peripheral directions, and most notably posteriorly from the rostrum and medially from just behind the orbits (Figure 2C, D). In the CT sections the outer compact bone appears highly dense, approaching 3,000 Hounsfield units (water = 0; dark red in Figures 2, 5, and 6). Deep attenuation of density indicates that beam hardening inflates apparent density of the superficial dome, although the lack of such a gradient from the palate dorsally suggests that the outer dome did consist of dense compact bone. Lower-density compact bone lines vascular traces that exit onto the dome surface (Figure 2 C–E), differentiating them from surrounding cancellous bone. (These tubular structures do not increase in density superficially, again indicating that high Hounsfield values of the surrounding cortex do not solely reflect beam hardening.) Similarly dense compacta occur just deep to the outer dome, and as two internal bands probably representing earlier stages of dome development (green bands, Figure 2 C and D).

The frontal dome of *Cephalophus* displays stratification like that of *Stegoceras*, with a dense compact layer (zone 1) adjacent to the braincase, and cancellous and compact layers superficially (Figures 4 and 5). Inflation of densities from superficial beam hardening is unlikely, because Hounsfield values are similar for the floor and roof of the braincase. Trabecular size and density are more variable in the *Cephalophus* scan than in *Stegoceras* (Figures 4, 5, 6). Some *Cephalophus* trabeculae recall the neurovascular conduits seen in *Stegoceras*, but form a cancellous latticework of struts rather than traversing the entire dome. The high density of some regions of the *Cephalophus* dome recalls regression of trabeculae seen in some large adult pachycephalosaurs [7], although the *Cephalophus* dome lacks their uniformity of compact bone.

Similarly to *Cephalophus* and *Stegoceras*, a mid-sagittal cranial section of *Ovis canadensis* (Figure 6C) shows deep compact, cancellous, and superficial compact zones, but with an additional sinus region (zone 2a: Figure 6C). The deep zone 1 [7] and cancellous zones (2b in *Ovis canadensis*) are especially similar between the specimens. We predict that more mature specimens of *Ovis canadensis* will possess a thicker compact zone 3, as seen in this *Stegoceras* and other pachycephalosaur specimens.

The *Ovibos* crania display bone density patterns grossly similar to those in *Cephalophus* and *Stegoceras* (Figures 7, 8, 9), but with relatively larger cancellous regions. As in *Cephalophus* the frontals of *Ovibos* are superficially dense, although no denser than compact bone of the nasals and maxillae. Densities of compact bone are higher in the adult *Ovibos* (Figures 7 and 8) than in the juvenile (Figure 9). Beneath the apices of the horn sheaths, and in line with the occipital condyles, a superficial layer of dense compact bone overlies a deep and extensive region of cancellous bone contiguous with the endocranial cavity (Figure 8). This internal structure recalls zones 1 and 2 of pachycephalosaur domes [7]. Unlike in pachycephalosaur domes, the bone lining the endocranial cavity is not notably dense, and the superficial compact layer is much thinner. Frontoparietal sinuses and associated struts of bone occur primarily lateral and anterior to the apices of the horn sheaths (Figure 8). Struts run anteroventrally within maxillary sinuses that are sagittally in line with the tooth rows.

**Figure 10:**
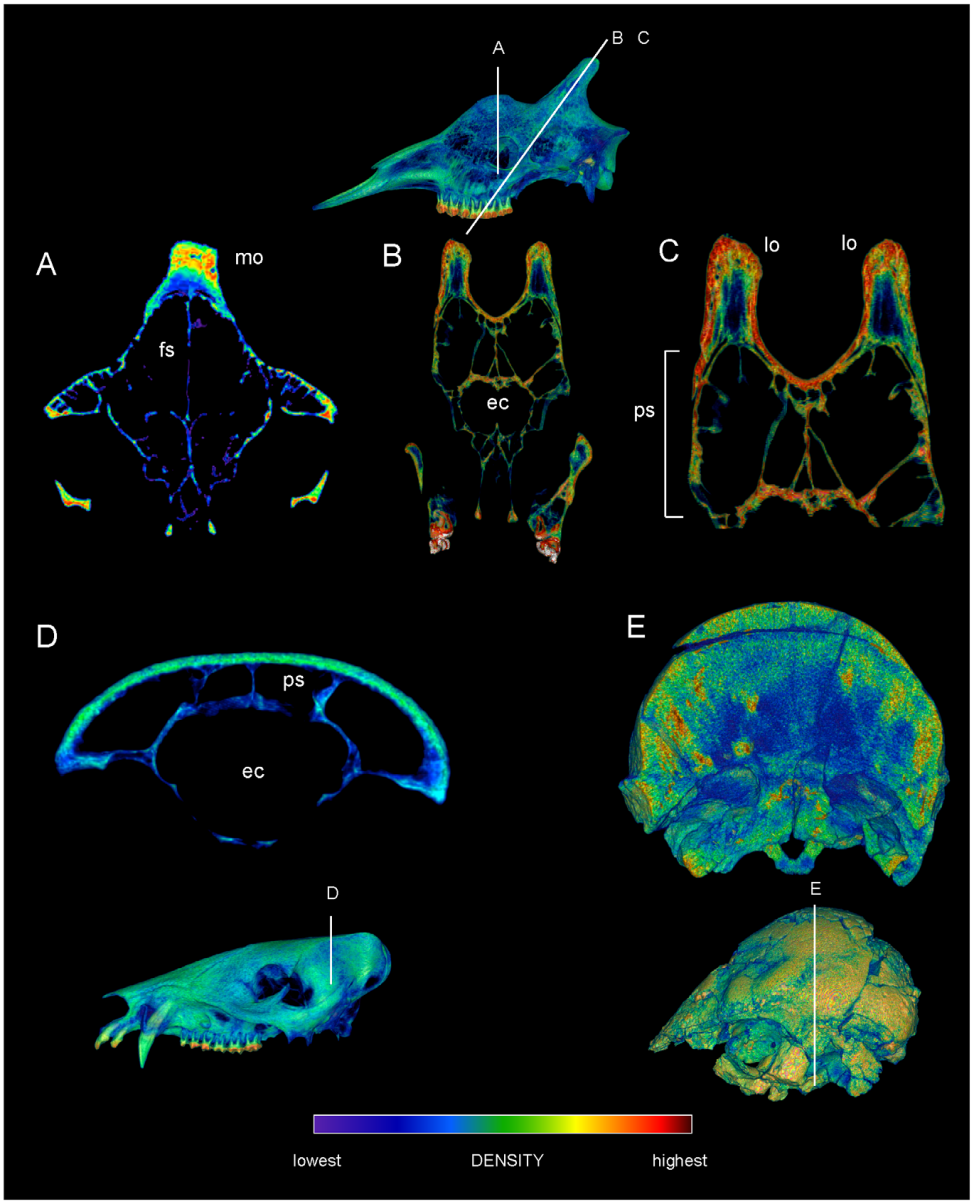
Cranial densities in *Giraffa* (TMM M 6815, UCMZ 1976.33), the peccary *Tayassu* (UCMZ 1975.279), and pachycephalosaur *Prenocephale* (GI SPS, field number PJC2004.8). CT sections through crania of comparative taxa, with slices mapped onto lateral renders of crania. A. *Giraffa camelopardalis* male (TMM M6815), transverse section through the region of a median ossicone. B. Oblique transverse section of *Giraffa camelopardalis* (UCMZ 1976.33) through the posterior ossicones. C. Enlargement of B focusing on the ossicones. The layering of densities in the giraffe ossicones resembles that in the dome of *Stegoceras validum* (Figure 6). D. Transverse section through the cranium of *Tayassu tajacu*, showing a non-cancellous skull roof over cranial sinuses. D. Section through the cranium of the pachycephalosaur *Prenocephale prenes* (GI SPS). Despite mineral inclusions (localized red and yellow areas) and CT artifacts, the scan shows dense superficial and cancellous deep regions within the dome. Abbreviations: ec = endocranial cavity, fs = frontal sinus, lo = lateral ossicone; mo = median ossicone; ps = parietal sinus.

**Figure 11:**
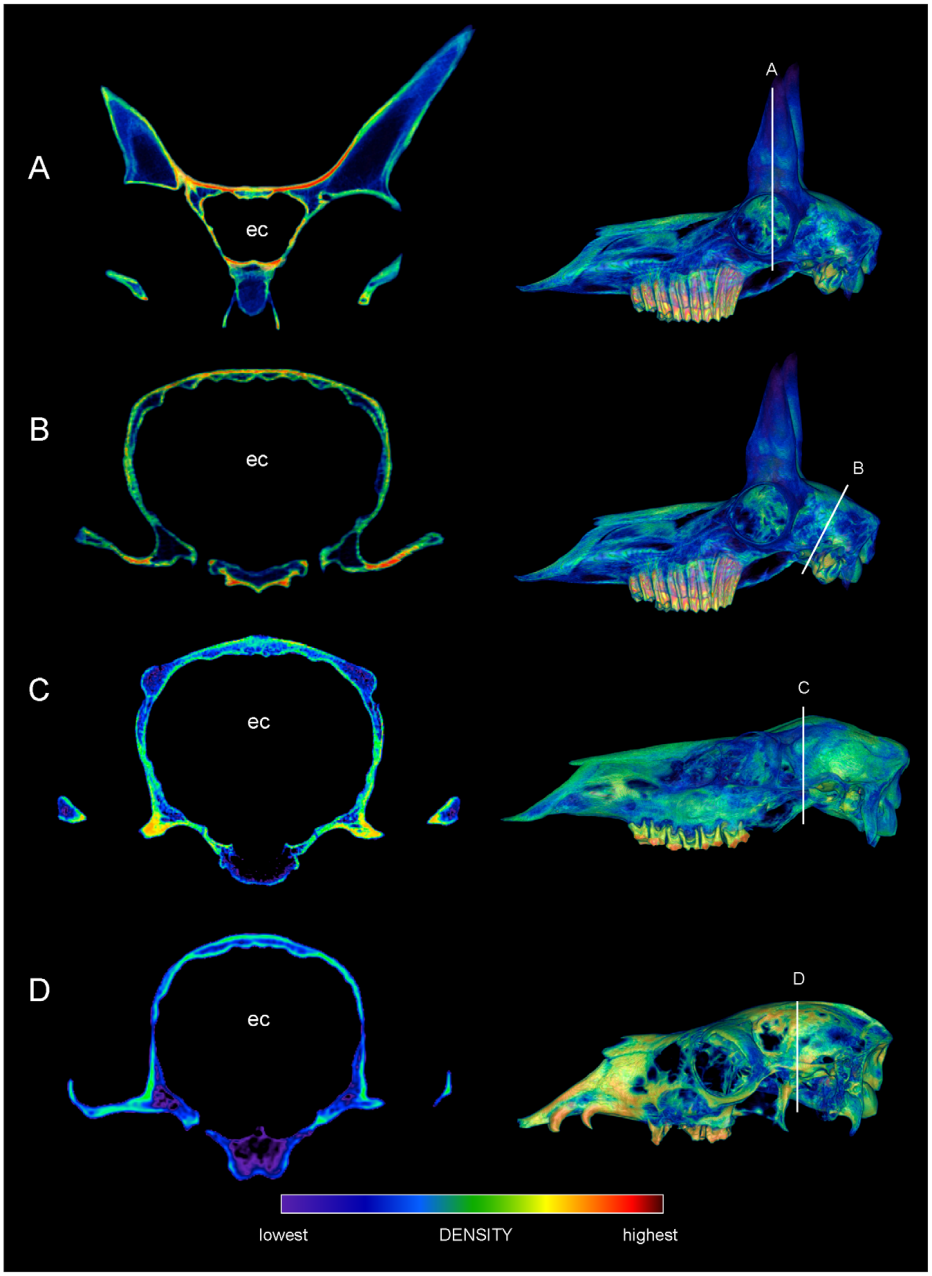
Cranial densities in the pronghorn (*Antilocapra*, UCMZ M 1989.61), elk (*Cervus* UCMZ M 1986.54), and llama (*Lama*, UCMZ M 1987.5). CT sections through artiodactyl crania, with insets depicting section locations on lateral CT reconstructions. A. Transverse section through the pronghorns and anterior braincase of *Antilocapra americana*, showing dense bone (bright red) where the pronghorns meet the skull roof but no cranial sinuses. B. Oblique section through the posterior cranium of *Antilocapra americana*. The lack of cranial sinuses is similar in both depicted regions. C. Transverse section through the cranium of *Cervus canadensis* reveals cancellous bone at the antler bases. D. Section through the cranium of *Lama glama* reveals a thin skull roof. These specimens' morphologies contrast with extensive cancellous bone and/or sinuses above the endocranium in head-striking artiodactyls *Ovibos* and *Giraffa*, and the pachycephalosaurs *Stegoceras* and *Prenocephale*. Abbreviation: ec = endocranial cavity.

The other artiodactyls vary substantially in distribution of cranial sinuses, trabecular bone, and compact bone (Figures 10 and 11). The *Giraffa* crania lack a thick cancellous region, and have more extensive cranial sinuses above the brain than do bighorn sheep (Figure 10A–C). Giraffe ossicones, however, have dense superficial compacta and deep region of less-dense compact bone (Figure 10B and C), as in the scanned domes of *Stegoceras* (Figure 6) and *Prenocephale prenes* (Figure 10E). The median ossicone of a large male giraffe (Figure 10A) has similar density distribution to that of the *Stegoceras* dome. However, its cortical region appears to lack long vascular canals running to the surface, and is more like a large *Pachycephalosaurus* dome in gross cross sectional appearance [7]. The peccary (Figure 10D) has cranial sinuses above the braincase, but its skull roof lacks a cancellous region. The *Cervus*, *Lama*, and *Antilocapra* specimens (Figure 11) lack extensive sinuses or trabecular regions above the braincase. The skull roofs of *Lama* and *Cervus* are thin in cross section; *Cervus* has some cancellous bone (particularly in the antler nubs), but this region is not particularly deep. The frontals of *Antilocapra* are thin between the flanking horn cores, but much denser than the equivalent region of *Cephalophus*. This inter-horn bone in *Antilocapra* is the densest seen in any of the artiodactyl crania, aside from bone of some auditory bullae,

### Finite element results and correlations with bone density distribution

**Figure 12:**
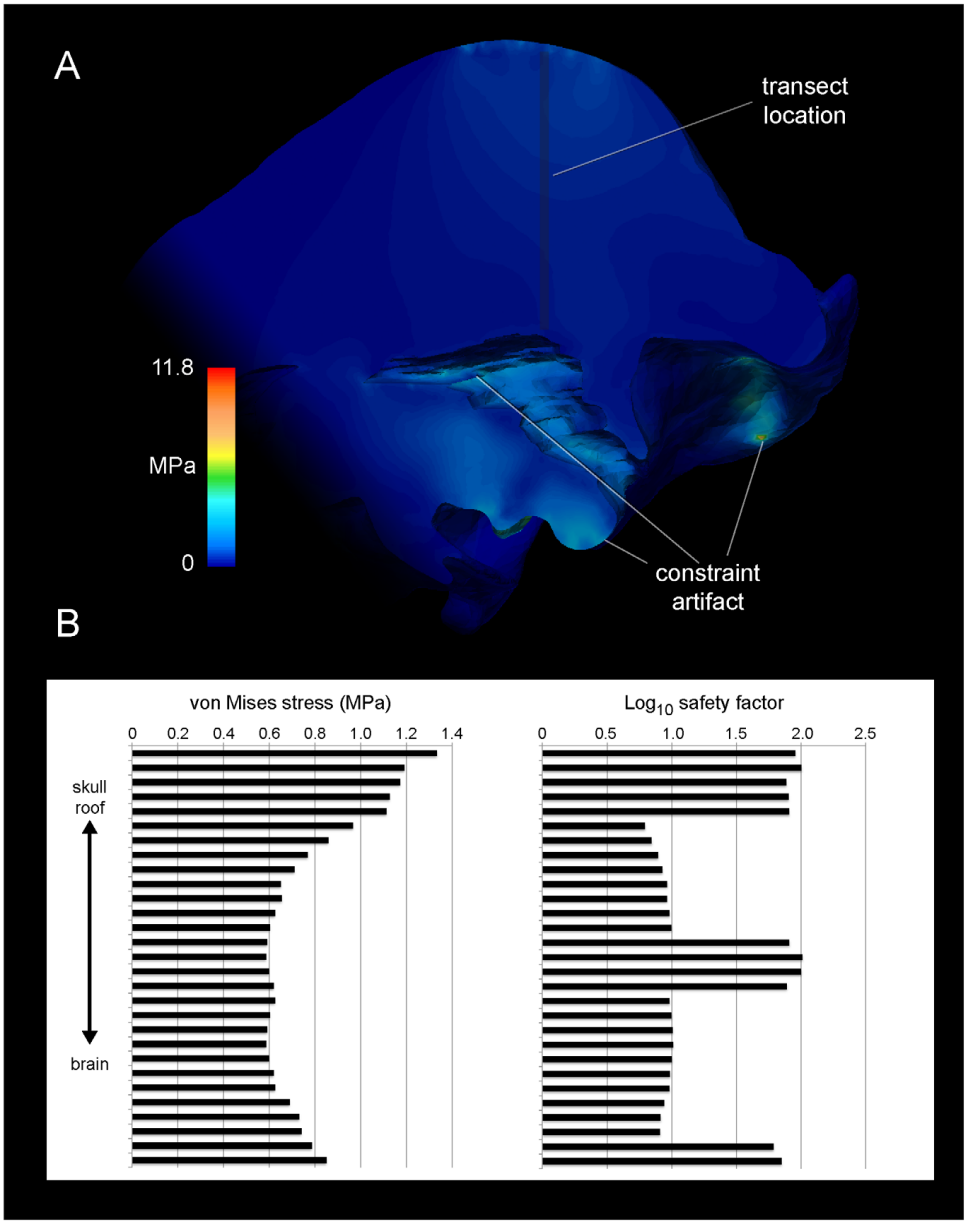
Stress and strain in the dome of *Stegoceras validum* (UA 2). A. Von Mises stresses (indicating closeness to yield) in a mid-sagittal section through the cranium of the pachycephalosaur *Stegoceras validum*. The highest stress within cancellous regions of the dome about 1 MPa, indicating a safety factor of 8–10 at the tested force. Stresses in compact bone surrounding the brain peak at 5 MPa, for a safety factor of 20–30. Constraints inflate stress artificially at the basal tubera and occipital condyle, and basicrainial and braincase stresses would be lower than depicted here. B. Von Mises stress and strain at 29 samples of a vertical transect through the dome. Strains are expressed in terms of safety factor: ultimate bone strain (0.6%) divided by the actual strain. Log_10_ values are used, because cortical safety factors approach 100 in some regions.

**Figure 13:**
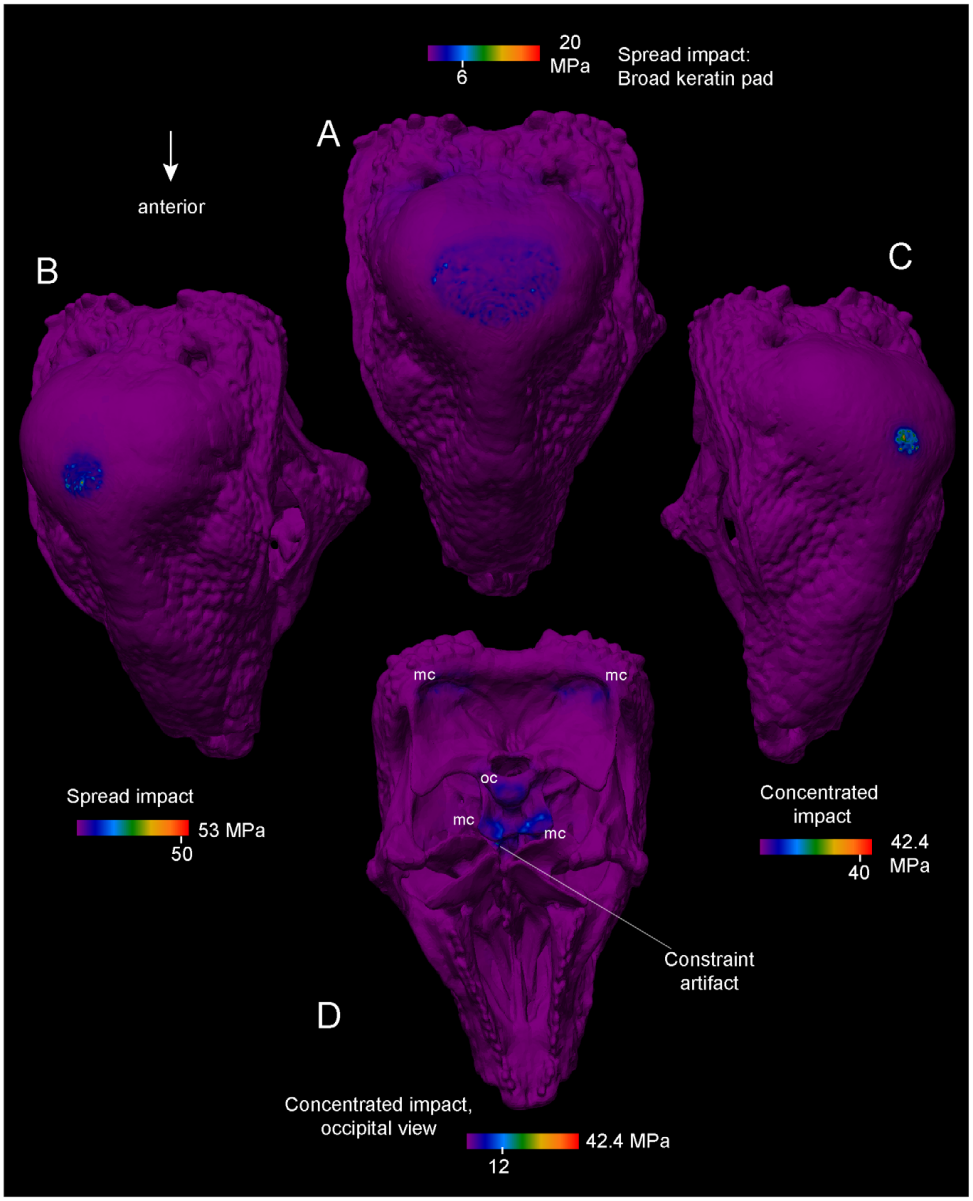
Effects of keratinous pad shape on head-butting stresses of *Stegoceras validum* (UA 2). For all simulations impact force is 1360 N, and stresses are von Mises values. The range and peak visible stress are noted in the color scales. A. Dorsal view of cranium. Force is distributed across a large surface area as a large keratin pad is deformed upon impact. Peak stress at the impact site is 6 MPa, and modal stress is 3 MPa. B and C. More concentrated impacts, simulating a thinner layer of keratin. D. Ventral view of stresses in impact C, showing occipital condyle (oc) and muscular constraints (mc). The detail level of this model (2 million elements) increases chances of artificially high stress at near-singularities, such as when force is applied to the edges of neurovascular canals.

***Stegoceras validum*** **(UA 2).** Figures 12 and 13

Stresses in the *Stegoceras* dome diminish rapidly deep to the area of loading (Figure 12). When force is applied to a broad area (simulating a spread of stress from a keratin covering), stress is lower and more diffuse (Figure 13). Strain energy is slightly higher in the cancellous internal region of the dome than in the compact periphery, but stress is so low that the increase in strain is apparent but minimal. Even in the most diffuse cancellous regions of the dome, strains never reach ultimate strain of cancellous bone (0.52–1.21% [15]; Figure 13B). With cancellous bone parallel to impact force assigned an elastic modulus of 1 GPa, safely factors are 5–10 at the 1360 N force of a simulated impact.

The high-resolution *Stegoceras* model (Figure 13) has more singularity artifacts than other models, but is still informative about force transmission through varying keratin pads. Stresses peak artificially at the occipital condyle constraint (up to 52 MPa) and never exceed ultimate or yield stress of compact bone, yet peak magnitudes vary with loading and constraint. The highest stresses at the apex of the dome are 46 MPa for concentrated impacts, and 8 MPa for cap-distributed impacts, at artificial singularities on the edge of a neurovascular canal. Deep to these artifacts, peak stresses are 1.5–2 MPa, similar to those seen in Figure 12. Stresses are relatively higher ventral to the brain cavity than dorsal to it, and as in the artiodactyls stresses are highest at the condyle or points of muscular constraint. Moderate strains in cancellous bone of the condyle and condylar neck indicate some cushioning effect [1]. However, when the models are also realistically constrained by dorsal neck muscles, stresses diminish at the occipital condyle and the floor of the endocranial cavity. Stress and strain are lower above the endocranial cavity in *Stegoceras* than in *Ovibos*.

**Figure 14:**
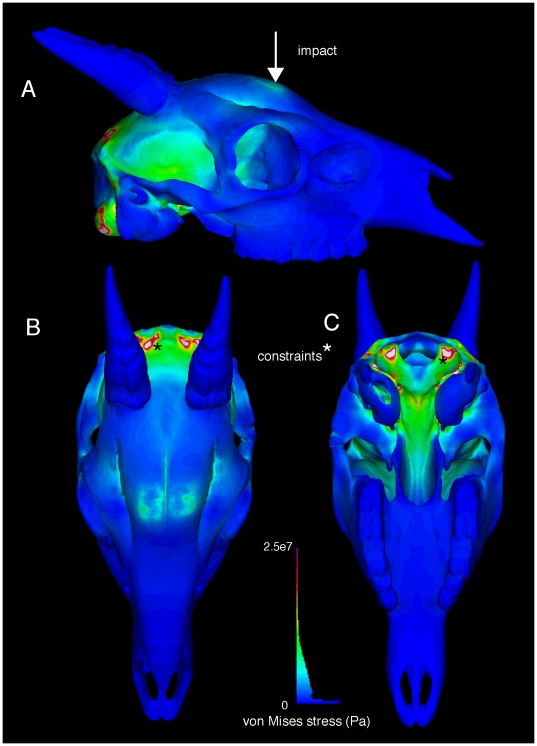
External views of finite element stress in the duiker *Cephalophus leucogaster* (AMNH 52802). Von Mises stresses of a 1360 N impact are depicted in lateral (A), dorsal (B), and ventral (C) views. Note artificial clipping occurs at the constraints. Higher stress occur at the impacts and around the brain than in *Stegoceras* (Figure 12), for the same collision force. The histogram depicts color coding for stress magnitudes, and the proportion of elements experiencing given levels of von Mises stress.

**Figure 15:**
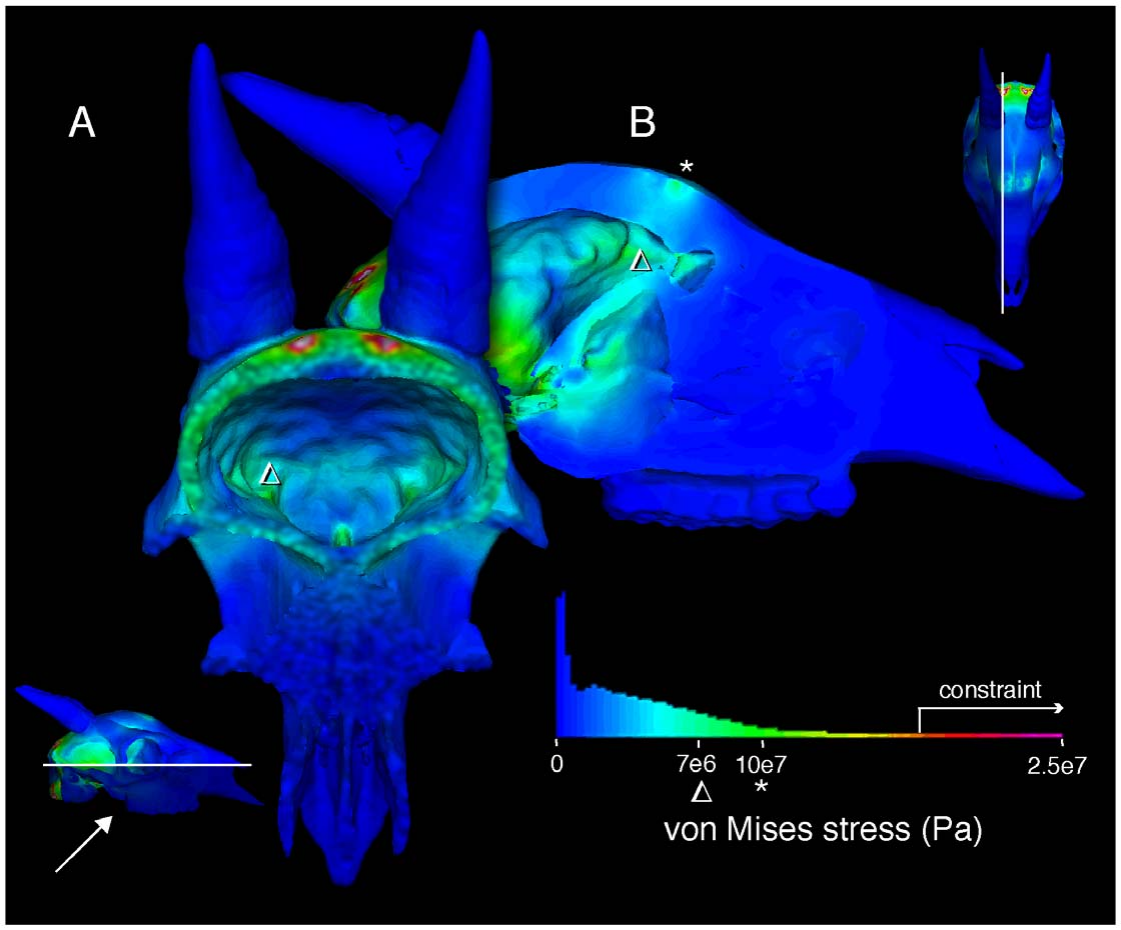
Internal stresses in the cranium of the duiker *Cephalophus leucogaster* (AMNH 52802). Internal von Mises stresses are evident in posteroventral oblique (A) and lateral (B) views, through sections shown in respective insets. Impact stress diminishes from superficial to deep (B), but greater stresses occur at on the internal surface of the braincase than in *Stegoceras*. The histogram reflects the relative number of elements at different stress magnitudes.

***Cephalophus leucogaster.*** Figures 14 and 15

Expected from its less voluminous dome, stresses in *Cephalophus* deep to the point of impact were higher than in *Stegoceras* for the same impact magnitude, despite the animals' similar basal skull lengths. Von Mises stresses decline precipitously from the impact towards the brain, falling from 10 MPa to 7, but the magnitudes remain higher than in *Stegoceras*. Two peaks of stress occur on the roof of the endocranium ventral to the dual impact sites (Figure 15 B), and overall stress of bone surrounding the brain (Figures 14, 15) is higher than in *Stegoceras*. The highest stress occurs at the constraints, and this stress does not diminish as markedly as in *Ovibos* or *Stegoceras*. Stress magnitudes in *Cephalophus* are much lower than in the flat-headed *Lama*, even when corrected for differing force magnitudes.

**Figure 16:**
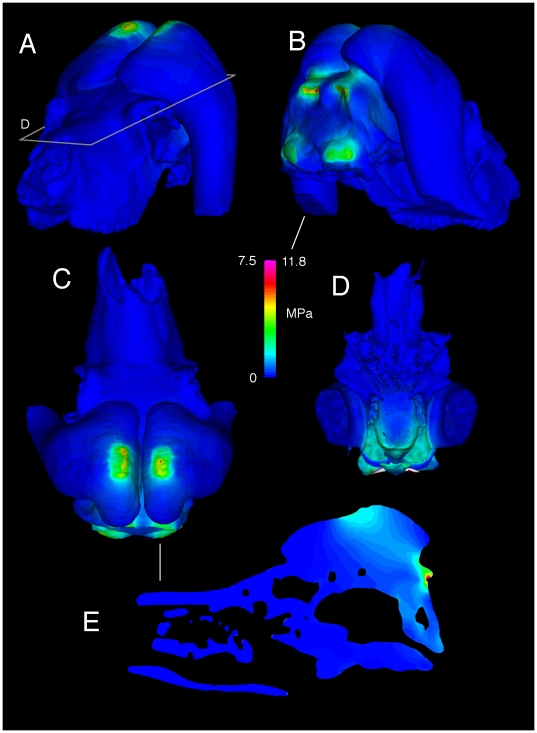
Finite element stresses in the musk ox *Ovibos moschatus* (UCMZ M 1978.1.92). Von Mises stresses for *Ovibos moschatus*, in anterior oblique, posterior oblique, and dorsal view (A–C). D. Ventral view into the braincase (sectioned at the plane shown in A), showing high stress posteriorly. The highest stresses occur at the site of impact and (artificially) at muscular constraints (D); note the different color scale for stresses. E. Sagittal section (location marked in C) shows higher stresses channeled away from the endocranial cavity, in line with the posteroventrally directed impact force.

***Ovibos moschatus*** **(UCMZ M 1978.1.92).** Figure 16

Stresses at the impact sites are higher in *Ovibos* than in *Cephalophus*, but both stress and strain diminish more rapidly deep to the force application. Stress and strain are higher in struts traversing the parietal sinus than in struts less in line with the impacts. Compact bone of the skull roof experiences low stress. Expected from its lower elastic modulus, cancellous bone over the brain and especially in the occipital condyles experiences higher strain than compact bone. The dorsal surface of the endocranial cavity experiences lower stresses and strains than in any of the other artiodactyls (Figure 16 D), but is higher than in *Stegoceras*. High stresses propagate through the keratin pad towards the muscular constraints (Figure 16 E).

**Figure 17:**
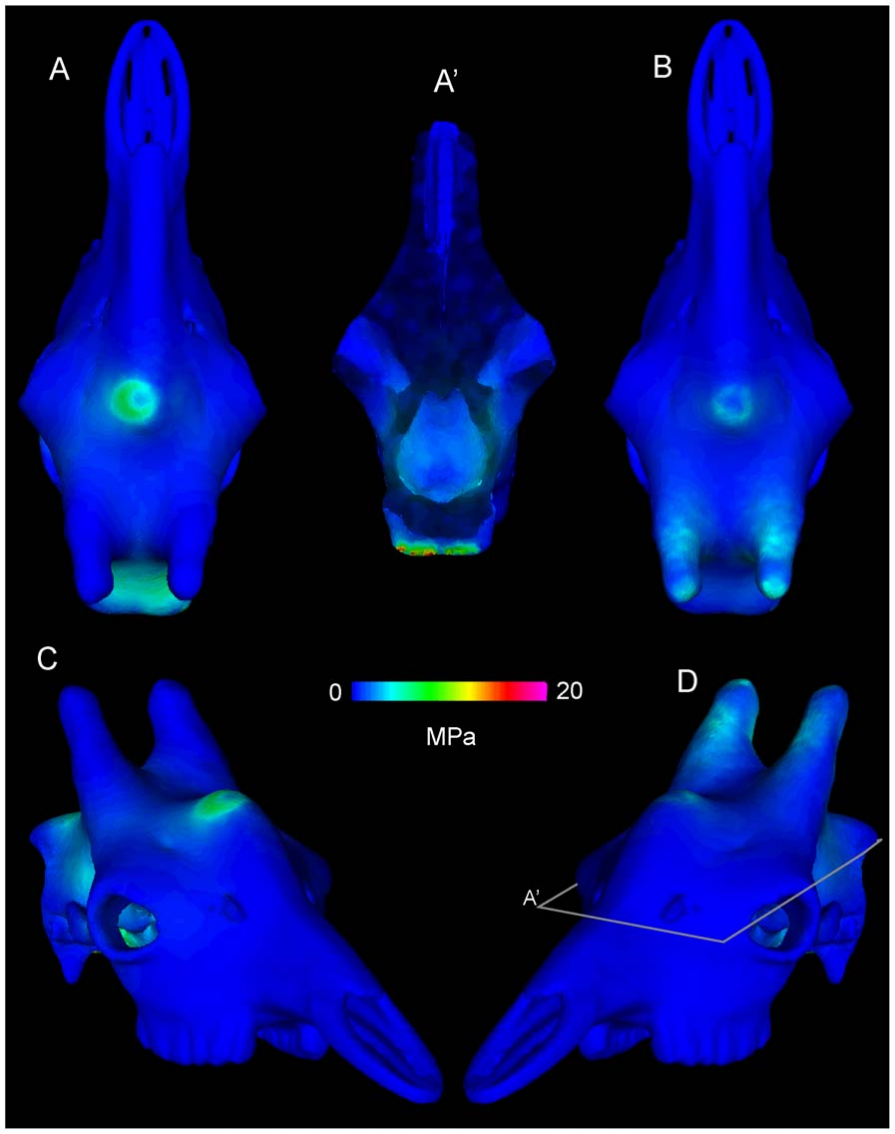
Finite element results of two head-strike simulations in the giraffe (UCMZ 1976.33). Von Mises stresses in *Giraffa camelopardalis*, with the cranium in dorsal A, B) and oblique (C, D) views. A′ is a ventral view of a coronally-sectioned cranium (at the plane in D), looking up into the endocranial cavity. A, A′, and C depict vertical impacts through the median ossicone and frontal sinus. Note comparatively high stresses at the posterior muscular constraint and substantial stress in the endocranial cavity compared with *Ovibos* (Figure 11 D), yet more localized stress than in *Antilocapra* (Figure 13). Peak stresses are lower when the force is spread over all three ossicones (B and D), suggesting that such impacts are more favorable to the animals.

***Giraffa camelopardalis*** **(UCMZ 1976.33).** Figure 17

Ossicones of *Giraffa* experience high stress relative to the impacting structures in *Ovibos*. Stress is also greater in struts within frontal sinuses of *Giraffa* than in similar struts of *Ovibos*. Stress and strain are low in compact bone over the brain cavity, but substantially higher in impacts to the median ossicone than when all three are loaded (Figure 17). This differs from the condition in indirect, horn-impact loads in *Capra* [1], which also has a sinus and struts beneath points of impact and most of the brain posterior to it. Because the *Giraffa* model created in Mimics® has an artifact of thickened intrasinus struts for proper volumetric meshing, in life stress and strain would be higher in these structures. However, the applied force (Table 1) appears higher than likely.

**Figure 18:**
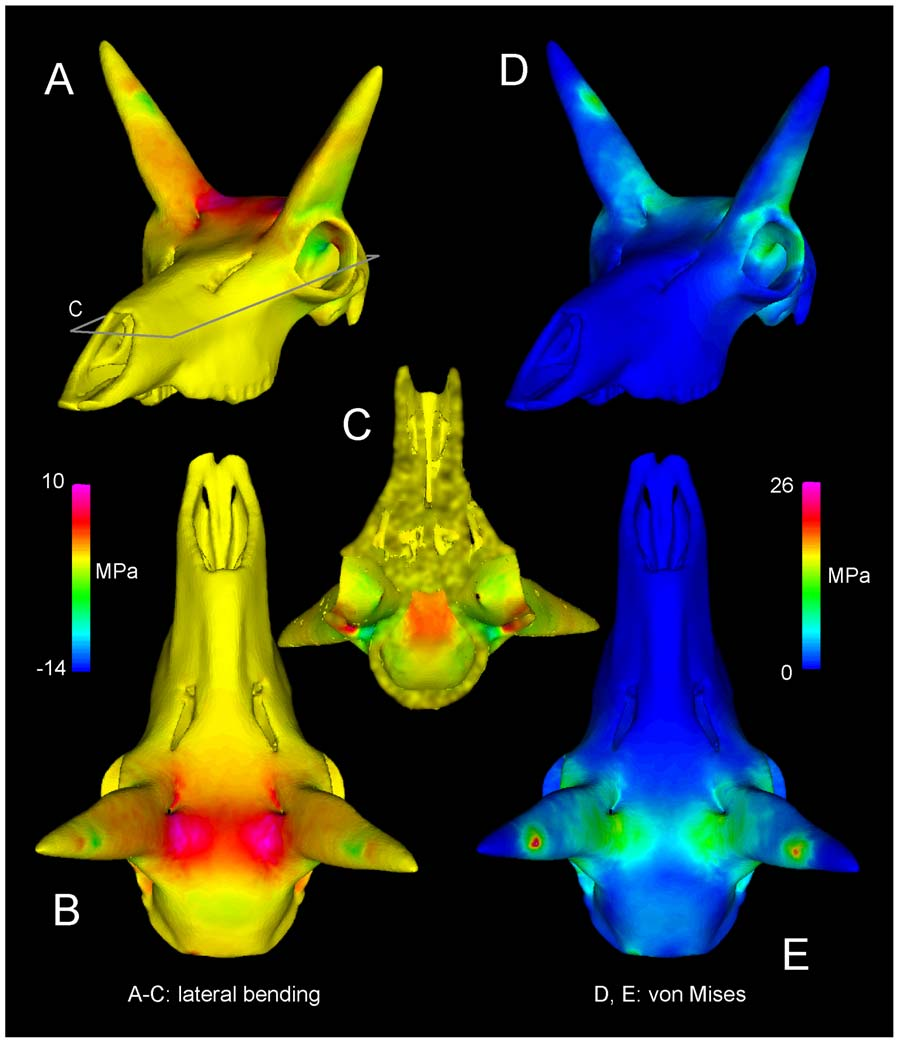
Head-strike stresses in the pronghorn (UCMZ M 1989.61). Stresses in *Antilocapra americana*, primarily from mediolateral bending (A–C) and consolidated as von Mises stress (D, E). C is a ventral view of the cranium sectioned in the plane shown in A. Relatively high tensile stresses occur at the base of the pronghorn cores (A, B) and roofing the endocranial cavity (C), where CT reveals dense compact bone (Figure 8).

***Antilocapra americana*** **(UCMZ M 1989.61).** Figure 18

Stresses in *Antilocapra* differed greatly from those of the other specimens (Figure 18). The horns display tensile stress medially and compression laterally, expected given that the impact would induce bending loads. The frontals at the base of the pronghorns experience high stress but not particularly high strain, yet the skull midline between the pronghorns displays high strain energy and high tensile stress in a complex pattern. Stress is high at the occipital constraints, but not as high as in the other artiodactyls.

Tensile stresses induced by lateral bending of the horncores predominate in *Antilocapra* (Figure 18). Although anteroposterior peak stresses are higher at the constraints, the frontals experience 5–10 MPa mediolateral tensile stress, and the base of the horncores −7 to −14 MPa compressive stress, over large areas. Locations of high stresses correspond to dense compact bone of the pronghorn cores and the frontals. Complex tensile stresses in the skull roof occur at the interfrontal suture (Figure 18), where bone is slightly less dense than that lateral to it. Cancellous bone between the pronghorn cores and brain cavity may experience tensile and compressive strains from lateral bending of the cores. Peak von Mises stresses are 26 MPa, for a safety factor of four to five.

**Figure 19:**
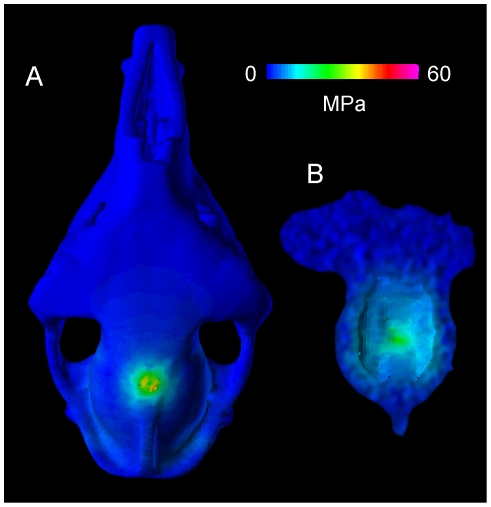
Simulated head-strike stresses in the llama (UCMZ M 1987.5). Von Mises stress from a simulated head impact in *Lama glama*, an artiodactyl that does not fight in this manner. A. Dorsal and B. ventral endocranial views depict higher stresses that occur at yield in cancellous bone orthogonal to the impact.

***Lama glama*** **(UCMZ 1987.5).** Figure 19

The *Lama* model experienced high bending stresses and strains, with primarily compressive stress on the skull roof and tensile stress on the dorsal surface of the endocranial cavity. The latter stresses are particularly high compared with those in the other examined taxa, despite artificial thickening of the model necessary for successful FE meshing. Peak von Mises stress reached 60 MPa for a distributed impact (Figure 19), and 120 MPa (which would chip the bone) for force applied to the parietal crest. These results are consistent with both structural and material characteristics of the *Lama* cranium. The skull roof is thin in the *Lama*, as in *Antilocapra*, but cranial bone densities and elastic moduli are lower. As with the *Giraffa* model, the *Lama* geometry is slightly inflated for solid meshing and stresses would be higher in realistically thinner bone.

### Recursive partitioning situates pachycephalosaurs among head-butting taxa

**Figure 20:**
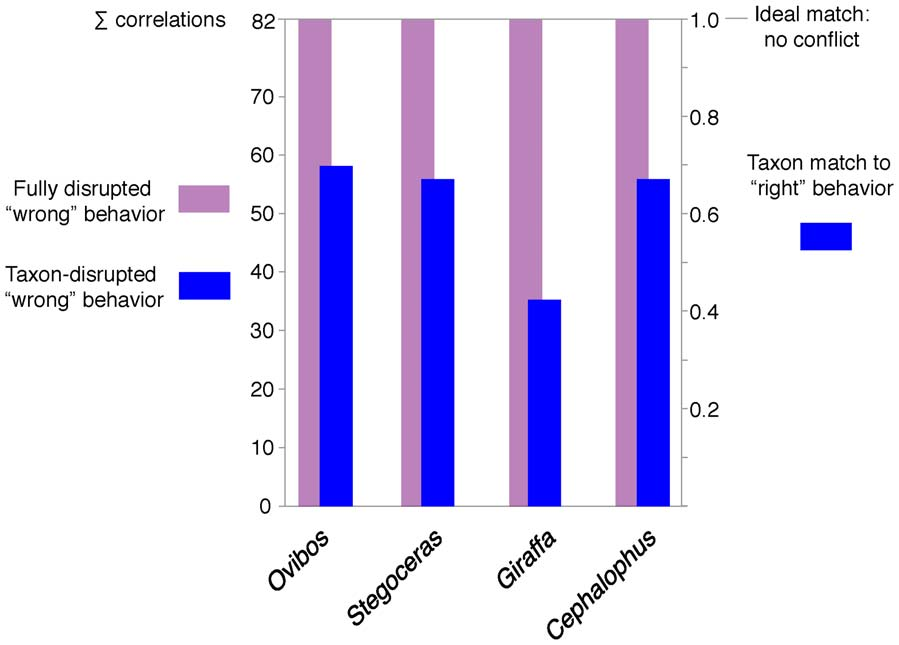
Strengths of behavior-morphology correlations for selected taxa. Correlate disruption values from Table 3, for incorrectly assigned behaviors in modern taxa and for *Stegoceras validum* when hypothesized as not head-butting. ∑ correlations values on the left indicate how much incorrect assignment perturbs the original correlations, and the scale on the right indicates how well the animals' morphology fits their “correct" behavior. The pachycephalosaur *Stegoceras* has a strong affinity with its hypothesized head-butting behavior, while the giraffe's lower score indicates ambiguous correlations between its morphology and behavior.

Figure 20 and Table 3 show strengths of correlation between cranial functional morphology and agonistic behavior (Table 2), and probabilities that taxa are correctly assigned to behaviors given their suites of morphology. The presence of extensive cancellous bone, dome versus flat head shape, and the size of neck muscle attachments have high correlation with behavior, whereas density of compact bone correlates less strongly.

**Table 2:** Possible categories and classes for recursive partition analysis.

			Morphologial categories				
		Compacta					
Taxon	Extensive trabeculae	Layer thickness	Density	Large struts	Surface vessels	Head shape	Neck muscles
*Stegoceras*	yes	thick	dense	struts	yes	domed	broad
*Ovibos*	yes	intermed.	dense	struts	yes	domed	broad
*Giraffa*	no	thick	dense	struts	no	spiked	narrow
*Lama*	no	thin	sparse	no struts	no	flat	narrow
*Tayassu*	no	thin	sparse	no struts	no	flat	narrow
*Antilocapra*	no	thin	dense	no struts	no	flat	moderate
*Ovis*	yes	intermed.	dense	struts	yes	domed	broad
*Synceras*	yes	intermed.	dense	struts	yes	domed	broad
*Buceras*	yes	thick	dense	struts	yes	domed	broad
*Equus*	no	thin	sparse	no struts	no	flat	narrow
*Cephalophus*	yes	thick	dense	struts	no	domed	broad

	Biomechanical categories		Behavioral classes	
	Braincase strain	Stress dissipation	Specific behavior	Agonism
*Stegoceras*	low	maximum	head strikes	yes
*Ovibos*	low	maximum	head strikes	yes
*Giraffa*	low	intermediate	body blows	yes
*Lama*	high	poor	no head butting	no
*Tayassu*			no head butting	no
*Antilocapra*	high	poor	no head butting	yes
*Ovis*			head butting	yes
*Synceras*			head butting	yes
*Buceras*			head butting	yes
*Equus*			no head butting	no
*Cephalophus*	low	intermediate	head butting	yes

Biomechanical categories can be included in RPA, but missing data gives more ambiguous results. Specific behavior and Agonism) are alternate choices for behavioral class. For layer thickness, “intermed" refers to intermediate compacta thickness. “Thick" for the giraffe refers to compact bone of the ossicones.

**Table 3:** Correlate disruption of recursive partitioning for selected taxa.

	“Correct"	*O.m.-*no	*S.v.*-no	*G.c.*-yes	*C.l.*-no
Cancellae	15.16	4.75	4.75	9.41	4.75
Cmpcta. thickness	9.41	2.28	4.57	4.18	4.57
Cmpcta. density	6.16	0.75	0.75	1.6	0.75
Struts	9.41	2.28	2.28	4.18	2.28
Vasculature	9.75	2.52	2.52	6.78	2.52
Head shape	15.16	4.75	4.75	9.41	4.75
Neck muscles	15.16	4.75	4.75	9.41	4.75
**G^2^ sum deviation**	0	58.13	55.84	35.24	55.84

The “Correct" G^2^ values are those for maximum correlation between behaviors and traits in Table 2. All other values are correlations when taxa are assigned to a different category than in Table 2. *Ovibos moschatus* (*O.m.*), *Stegoceras validum* (*S.v.*), and *Cephalophus leucogaser* (*C.l.*) are changed to “no" head-butting, and *Giraffa camelopardalis* (*G.c.*) is designated as head-butting. G^2^ sum deviations are how much the taxa disrupt the additive strength of correlations (the sum of the correct values minus the sum of disrupted values). Higher disruption values suggest better original assignments of behavior.

Correlate disruption suggests that *Stegoceras* has a high head-butting probability when included among extant taxa. Classifying the head-butting *Cephalophus* and *Stegoceras* as non-combatants reduces likelihood ratio chi-square values (G^2^) by the same amount; the morphology of *Ovibos* is a slightly better fit. In contrast, *Giraffa* disrupts G^2^ values less than the other taxa, indicating that giraffe morphology is better classified outside that of the animals that strike their heads together directly.

## Discussion

Extant combative ungulates vary in adaptations for head-butting, and pachycephalosaurs possessed a combination of their respective traits. In extant ungulates, recursive partition analysis shows strong correlations between head-butting and cranial morphologies that also occur in *Stegoceras*. Both *Ovibos* and *Giraffa* have dome-like structures that dissipate force, yet in *Ovibos* cancellous bone lies over the brain as compliant protection, and in the giraffe the point of impact lies anterior to the brain cavity and above struts of the frontal sinus. The *Antilocapra* model experiences tension in the thin skull roof. The domes of *Stegoceras* and *Prenocephale* resemble *Ovibos* heads in general shape and occurrence of cancellous bone, and domes of *Cephalophus leucogaster*, *Ovis canadensis* and giraffe ossicones in stratification of compact and trabecular regions.

The closest morphological matches of pachycephalosaur domes are the domes of *Ovis canadensis* and especially *Cephalophus leucogaster* (Figure 7), despite the presence of sinuses and/or horns in the artiodactyls. Bighorn rams famously collide with their horns by rearing and falling towards each other, which produces loud reverberations. However, they also charge and collide with the tops of their heads between the horns, a direct head-butting behavior similar to head strikes suggested for *Stegoceras*.

Recursive partitioning suggests that *Stegoceras*'s morphology fits head-strike behavior comparably to the duiker and musk ox. More taxa and biomechanical results would better evaluate strengths of character/behavior associations, and improve on our application of the method. However, strong correlations of head butting with features common to this *Stegoceras* specimen and fighting artiodactyls suggest behavioral and biomechanical commonalities.

Biomechanical FEA results of this study corroborate the interpretations of Farke [1] for artiodactyls and Snively and Cox [8] for pachycephalosaurs. The large osseous dome of *Stegoceras* is more effective at spreading force than the lower domes of *Cephalophus*, and the keratin pad of *Ovibos* better absorbs strain [8], [10]. However, the *Cephalophus* FE model experienced much lower relative stress than did the thin-skulled lama, suggesting that thick, solid frontals suit duikers well for head-butting [1], [5], [6]. Although we were unable to vary material properties in the *Cephalophus* model, we can predict lower strains close to the endocranium than in *Stegoceras*. Higher densities and larger struts of cancellous bone (Figures 4, 5, 6) in *Cephalophus* would result in higher stiffness, lower deformation, but less absorption of impact energy than in the pachycephalosaur. High cancellous strain in *Ovibos* corroborates Farke's [1] FE results with a simulated trabeculae-filled frontal sinus, and his prediction that copious struts within Cape buffalo and bighorn sheep crania would absorb shock of vigorous head-butting. Both Farke's and the current study strongly parallel Maity and Tekalur's findings for bighorn sheep [10], which will further guide evaluations of strain and material effects on combative behaviors.

Similarly to Snively and Cox's [8] simple geometric models, the CT based *Stegoceras* FE model experiences low cortical strain, higher (but still low) cancellous strain, and dramatic reduction of stress distal to the impact on the dome surface. The cortical portion of the dome experiences peak stresses of 8–46 MPa, below their ultimate levels (180–200 MPa in compression: [15]; incorrectly set at 300 MPa in reference [8]). For its cortical bone to reach ultimate stress and strain [15], this *Stegoceras* would have to impose 5–10 times the tested force on its dome. The highest peak stresses occur at the edges of neurovascular canals, where the mesh has artificially sharp angles; lower cortical stresses adjacent to these artifacts represent the more probable life condition. Cancellous stress and strains peak at 1 MPa and 0.02%, below failure levels (6–12 MPa; 0.2–1.21% strain [11], [16], [17]) even in low density regions of the dome. This suggests that failure of the entire dome was unlikely even in younger pachycephalosaurs with primarily cancellous bone, despite their diffuse, apparently fragile trabeculae. Radiating trabeculae In older adults with primarily compact bone, strain and displacement near the brain would be negligible, similar to results from Farke's [1] simulations of an artificially solid dome in *Capra*.

The dome of this specimen of *Stegoceras* was structurally capable of dissipating force of impacts against solid objects, more so than skull roofs of artiodactyls known to head-butt at high forces. However, low dome vascularization in large individuals [7], and presumably reduced healing ability and supply to a keratin pad, argue against head-head combat in older pachycephalosaurs [7], [8]. Examination of older *Cephalophus leucogaster*, which head-butt despite dense domes and lack of a large keratin pad above the point of impact, will test this interpretation. Apparent beam hardening in the *Stegoceras* scans highlights the necessity of physical sectioning and histological examination [7] to check CT densities in fossil vertebrates. Regardless, dome strengths of large, old and young pachycephalosaurs must be examined with the present or similar methods, to assess impressions based on histology, CT data, and even FEA of the current specimen.

The scanned adult *Stegoceras* shares morphological correlates of head-butting with extant artiodactyls. A deep cancellous region beneath a point of impact occurs in the largest head-striking artiodactyls, including *Ovibos* and *Giraffa*, and we predict this zonation in CT scans of smaller head-striking forms. *Stegoceras* lacks pneumatized frontal sinuses present in *Giraffa* and *Ovis canadensis*. However, compact bone surrounding vascular traces in *Stegoceras* forms tubular struts that traverse the dome, recalling the struts within cranial sinuses of ungulates. These neurovascular canals open onto the skull surface, a condition Hieronymus et al. [2] identify as a correlate of cornified pads (as in *Ovibos*) covering the crania of head-butting artiodactyls and hornbills. The struts in *Stegoceras* are interpretable simultaneously as vascular conduits feeding development of a keratin covering [2], and as structural braces analogous to struts in artiodactyl cranial sinuses. Both biomechanically and developmentally the tubes serve as potential correlates for head butting capability in pachycephalosaurs. This hypothesis will be falsified if the struts are shown to be loosely anchored or little affected by overall impact stress.

Our findings, and those of Farke [1] and Maity and Tekaur [10], point to other such predictions of functional morphology in known and putative head-butters, testable through CT and FEA. CT of tapinocephalid synapsids will likely show struts within sinuses similar to those imaged here in *Tayassu*, and present in head-butting suids that Barghusen [19] identifies as behavioral analogs. When combined with conceptual advances for evaluating structures unknown in modern fauna [20], finite element modeling will further advance hypotheses of behavior in fossil animals beyond anatomical and mechanical intuition.

### Future directions: transient analyses and energy dissipation

The forces applied thus far to FE models of pachycephalosaurs, *Cephalophus*, *Capra* [1], and *Ovis* [10] are reasonable impact loadings [18]. However, FEA has approximated impact events with steady-state, linear static simulations. True collision simulations are unlikely to greatly alter stress and strain results, but will better enable analysis of energy dissipation by trabeculae. Trabeculae and larger struts angled relative to the impact force would be loaded in bending and be weaker than in compression, but would absorb strain energy better than struts parallel to the force [1], [15]. The role of solid struts or neurovascular conduits (seen in *Stegoceras*) would be possible to model using 2D analyses [8]. Characterizing how morphology contributes to energy dissipation has potential application to military, motorcycle, and sporting helmets designed to reduce injury [21], [22,] [23].

## Materials and Methods

### CT scanning, geometry reconstruction, and finite element meshing

Specimen numbers, lengths, and forces for included taxa are listed in Table 1; all University of Calgary specimens had been zoo animals. The cranium of a male bighorn sheep (*Ovis canadensis*) was sectioned mid-sagittally with a bone saw. Crania of *Stegoceras validum*, *Ovibos moschatus*, *Giraffa camelopardalis*, *Cervus canadensis* (elk), *Lama glama*, *Antilocapra americana* (pronghorn), and *Tayassu tajacu* (peccary) were scanned on a General Electric Lightspeed CT scanner (Canada Diagnostics Centre, Calgary, Alberta), at settings for diagnoses of bone pathology. The *Stegoceras validum* specimen was also scanned on a high-resolution x-ray CT at the University of Texas at Austin. *Ovibos* specimens represent a juvenile and an adult, as determined by examining tooth eruption patterns. A cranium scan of the duiker *Cephalophus leucogaster* was provided by Andrew Farke, also scanned on a General Electric Lightspeed medical scanner [5]. We also imaged a section through the median ossicone of a large male giraffe (Texas Memorial Museum TMM M6815, scanned by Timothy Rowe), from a CT sequence on the University of Texas, Austin Digimorph web site (http://www.digimorph.org/specimens/Giraffa_camelopardalis/skull/, accessed August 17, 2009). For anatomical comparison with *Stegoceras*, we examined scans of a large specimen of the Mongolian pachycephalosaur *Prenocephale prenes* (Geological Institute Section of Palaeontology and Stratigraphy GI SPS, field number PJC2004.8), then on loan to Philip Currie (University of Alberta).

Except for the male giraffe (TMM M6815), all CT data were in DICOM format. We used OsiriX® for 2- and 3D visualization of structure and density, to evaluate internal density distribution and guide reconstruction of finite element geometry and material properties. Densities were assessed primarily with the full-color NIH lookup table, which visualizes density gradations more clearly than do grayscale palettes. OsiriX® can section CT volumes in transverse, coronal, and sagittal planes. In other planes, we used the scissor tool to remove unwanted rendered bone, and produce sections approximately two mm thick. This was necessary for the *Prenocephale* specimen, which was rotated slightly out of anatomical neutral pose on the scanner bed, and for anteroventrally sloping sections of two artiodactyls. The latter angled sections are of an un-impacted control region through the braincase of *Antilocapra*, and lateral ossicones of the female *Giraffa* specimen.

We produced cranial finite element models of *Stegoceras*, *Cephalophus*, adult *Ovibos*, *Giraffa*, *Lama*, and *Antilocapra*. Mimics® (Materialise) facilitated construction of most 3D models (Figure 16) from density-based masks on individual CT slices, after methods of Arbour and Snively [24] and Bell et al. [25]. Internal spaces were automatically modeled from scans for the artiodactyls, but for the *Stegoceras* models, imaged matrix had to be removed manually within the cranial sinuses and endocranial cavity. The resulting models were saved as .stl surface meshes, and errors detected and corrected cyclically in the Mimics® remesher and Geomagic® Studio (Geomagic Inc.), for compatibility with Strand7® (Strand7 Pty Ltd) finite element analysis software. We used Avizo® (Visage Imaging) to construct the finite element model of *Cephalophus*. The surface mesh was refined initially with Avizo®'s RemeshSurface function, and remaining errors corrected with the software's surface editor. As with the Mimics®-based artiodactyl models, Avizo® yielded a high resolution tetrahedral model with larger struts and nasal conchae intact.

From these surface models and masks, we constructed two types of FE models. For the *Stegoceras*, *Antilocapra*, *Giraffa*, and *Lama* specimens, Strand7® and Mimics® produced error-free tetrahedral solid meshes, suitable for FEA, from a triangular surface mesh that included surfaces around internal cavities (Figures 11, 13, 14, 15, 16). For one model of *Ovibos*, a broken zygoma and circumorbital bone hindered tetrahedral meshing from its surface model. Instead of simplifying the modeled osteology, we produced voxel-based, hexahedral FE meshes using Mimics®, from density masks of both *Ovibos* and the hollowed-out *Stegoceras* CT scans. Incorporating all voxels results in millions of elements with prohibitive memory requirements and computation time. We therefore grouped voxels into larger sets, for meshes of approximately 200,000 hexahedra of varying shape (not just cubes) to better approximate original surface contours. This grouping retains anatomical details such as struts of bone, but results in a blocky external appearance in parts of the model. Even with varying element shape, a strictly hexahedral mesh is less “smooth" and accurate than a combination of hexahedral and tetrahedral elements, as Jasinoski et al. [26] constructed for dicynodonts. However, a large number of nodes in a hexahedral mesh ensures adequate resolution of results. We used high-resolution surface models to smooth the appearance of the FE mesh and visualize both mesh and external geometry.

An additional FE model of the *Stegoceras* cranium was created based on the Austin CT scan, which had twice the transverse resolution and 4.5 times the anteroposterior resolution of the original medical scan. A tetrahedral mesh of 2.2 million elements was created in Avizo®. This model became the primary one for analyses of the *Stegoceras* cranium.

### Material properties and kinematic constraints

**Figure 21:**
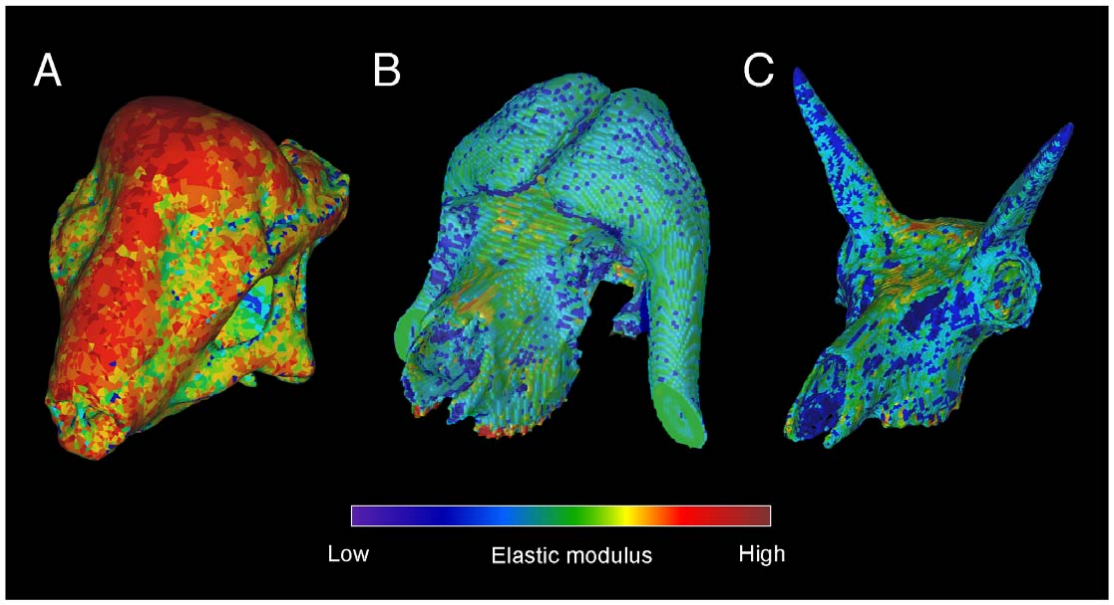
Finite element models with CT-based stiffness values. Elastic modulus indexed to CT Hounsfield values in (A) *Stegoceras validum* (UA 2), (B) *Ovibos moschatus*, and (C) *Antilocapra americana*. Moduli were assigned manually for *Stegoceras*, because CT beam hardening inflated some densities and fossilization obscured others. Modulus assignment for bone was automated in *Ovibos* and *Antilocapra*, and the pad in *Ovibos* assigned the modulus of alpha keratin.

We used Mimics® to assign material properties based on Hounsfield density values to most of the extant specimens (Figure 21), using procedures similar to those of Arbour and Snively [24] and Bell et al. [25]. With this method we assigned elastic modulus to bone in 18 discrete ranges of density, by the equation [25]:
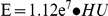
relating elastic modulus (E) and Hounsfield unit opacity (HU) from data of Hellmich et al. [27]. This equation yields higher elastic moduli than others, but better encompasses values in the upper end of the observed range [26], [28] and allows meaningful comparisons of overall morphological performance. Because keratin does not follow the same relationship, we manually assigned its properties (E = 3.9 GPa, ν = 0.28, ρ = 1300 kg/m^3^ [29]) to the keratin pad of the musk ox. The *Stegoceras* densities could not be fully automated (Figure 21) because the extent of permineralization was unknown. To densities above 2500 HU in *Stegoceras* we assigned properties of that density, under the assumption that beam hardening artifacts inflated values above those of the original compact bone. Cancellous bone in *Stegoceras* was assigned a conservatively low elastic modulus of 1 GPa. In cattle and humans [30], E = 0.5–4.5 GPa in dense cancellous bone like that seen in this *Stegoceras* dome. Mimics® was unavailable for producing density-stiffness assignments for *Cephalophus*, and its cranium was given a uniform density of compact bone (17 GPa [15]); the implications of this uniformity are discussed above.

To ensure that these structures would deform realistically under simulated head impacts, we constrained the models in two ways. Assuming transmission of force through the occipital condyles to the atlas, we constrained the condyles against translation and rotation. We also constrained the models along the rim of the nuchal crest after McHenry et al. [31], assuming offset forces restrained by neck muscles (m. transversospinalis capitis/m. complexus in *Steoceras* [32], [33], [34]; m. splenius capitis in the mammals). Muscular constraint at the basitubera in *Stegoceras* resulted in high stress artifacts ventrally, but did not affect stress levels in the dome.

### Forces and interpretation of structural performance

We applied compressive forces to apices of the horn sheaths in *Ovibos*, the top of the dome of *Stegoceras*, ossicones of *Giraffa*, the anterior parietal of *Lama*, and the medial surfaces of the horns in *Antilocapra*. Force applications are evident as dorsal concentrations of stress in Figures 12, 13, 19. Loads were applied to several areas of the *Stegoceras* model in separate analyses (Figure 13), because the size and spread of force through a keratin pad is unknown [8]. Two analyses were run on the giraffe, with forces respectively applied to the median ossicone alone, and to the median plus both lateral ossicones.

Magnitudes of stress and strain scale linearly with force magnitude unless a structure is greatly deformed. Stress and strain distributions are independent of force magnitude when force is applied to precise areas (and material is behaving elastically). In the absence of data on forces, it is reasonable to apply a unit magnitude to all FE models in a comparison, and directly scale stress and strain magnitudes when realistic forces are determined. However, we scaled forces to puts them in a biological context, and to open our assumptions to criticism. The baseline force for *Stegoceras* specimens was 1360 N, calculated for the similarly-sized pachycephalosaur *Homalocephale colathoceros* at a closing speed of 3 m/s [8]. Comparability of structural performance in animals dictates that forces are scaled to the subjects' sizes, ideally to surface areas for feeding comparison [35]. Considering their great variety of head shapes, we used a different scaling method for the artiodactyls. Their baseline force was 1088 N, calculated for *Capra* with a skull length of approximately 0.18 m [36], [1]. Assuming the same impact velocity and force proportional to skull mass, this force was then scaled to cube of the ratio of skull lengths for each artiodactyl versus *Capra* (Table 1). The force appears to be excessive for the giraffe, and caution is warranted for strict interpretations of its performance relative to other taxa. Because *Cephalophus* has a similar basal skull length to *Stegoceras*, we applied the same 1360 N to its cranium.

### Recursive partition analysis

Inferring behavior in fossil animals is possible by phylogenetic comparisons [37], [38], especially when pertinent behavior, morphology, and physiological response [34] converge in the taxon's extant phylogenetic bracket [38]. Phylogenetic inferences become less practical with more specific behaviors and a less constrained extant bracket [34], [38], and are inapplicable to pachycephalosaur head-butting. However, we can examine strengths of morphology/behavior correlation in extant taxa, and by inference in possible extinct analogs, by recursive partition analysis (RPA). The method was implemented using JMP® (SAS Institute, Inc.).

In RPA [2], strengths of correlation are proportional to the likelihood ratio chi-square values (G^2^) of correlation between a category (such as behavior) and a potentially influencing factor (such as a morphological feature or biomechanical result). We included comparative taxa whose morphological traits we could observe directly. These included specimens of the cape buffalo *Synceras caffer* which clash through the flat portion of their horns, the helmeted hornbill *Buceras vigil* which collide in flight with keratin-covered osseous domes [2], and the horse *Equus caballus* which do not engage in head strikes.

We chose not to include phylogeny as an influencing factor in recursive partitioning. Analogous morphology and behavior can arise in distantly related groups, yet behavior can differ between closely related clades even at the species level. Our application of RPA strictly assessed correlation between morphology and known or hypothesized behavior. Because behavior in an extinct taxon is usually unknown, phylogeny might unduly bias the strength of the taxon's behavioral assignment. An example would be bias towards head butting in a thin-skulled extinct bovid, if “Bovidae" is a trait that otherwise correlates well with ramming behavior. However, RPA results can be informative in later studies that optimize co-evolution of behavior and morphology onto known phylogenies.

To assess the strength of morphology-behavior correlation for individual taxa, we used correlate disruption as described in the introduction. Correlate disruption is the decrease in likelihood ratio chi-square values (G^2^) from the sum of “correct" G^2^ values (CD = ΣG^2^ correct-ΣG^2^ incorrect), which conversely indicates the fit of an animal to assigned behavior.
